# Supplementary figures and images for: A modified Agrobacterium-mediated transformation for two oomycete pathogens
Source: PLoS Pathog. 2023 Apr 21;19(4):e1011346. doi: 10.1371/journal.ppat.1011346 (PMC10156060; doi:10.1371/journal.ppat.1011346)

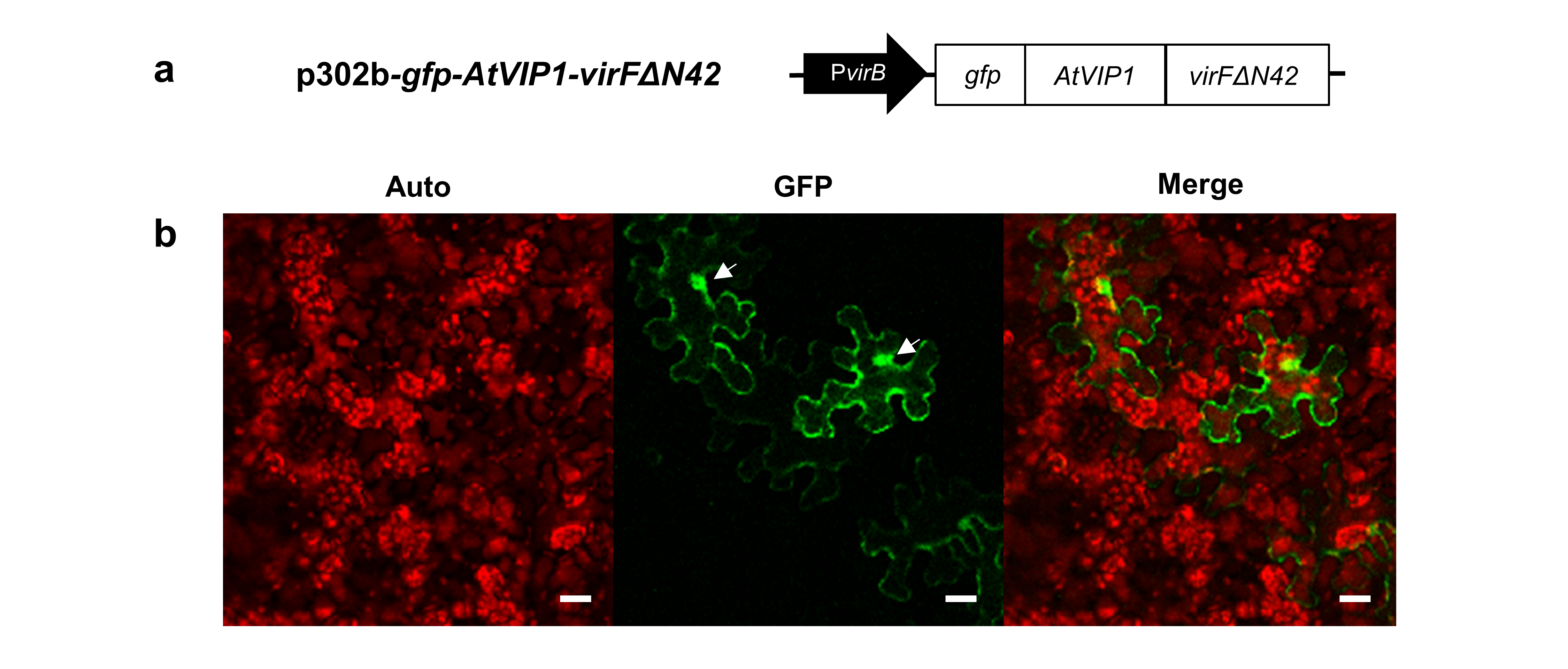

Supplement: S1 Fig — (a) The expression cassette used for translocating AtVIP1 fused with GFP (b) Confocal microscopy observation of N. benthamiana leaves infiltrated with A. tumefaciens EHA105 carrying the construct described in (a). Images were taken at 3 days post infiltration. Images are single confocal sections and are representative of images obtained in three independent experiments. White arrows indicate observed nuclei in tobacco cells. Scale bars = 40 μm. Three independent experiments were performed for each assay with similar results. (TIF) [file ppat.1011346.s005.tif]

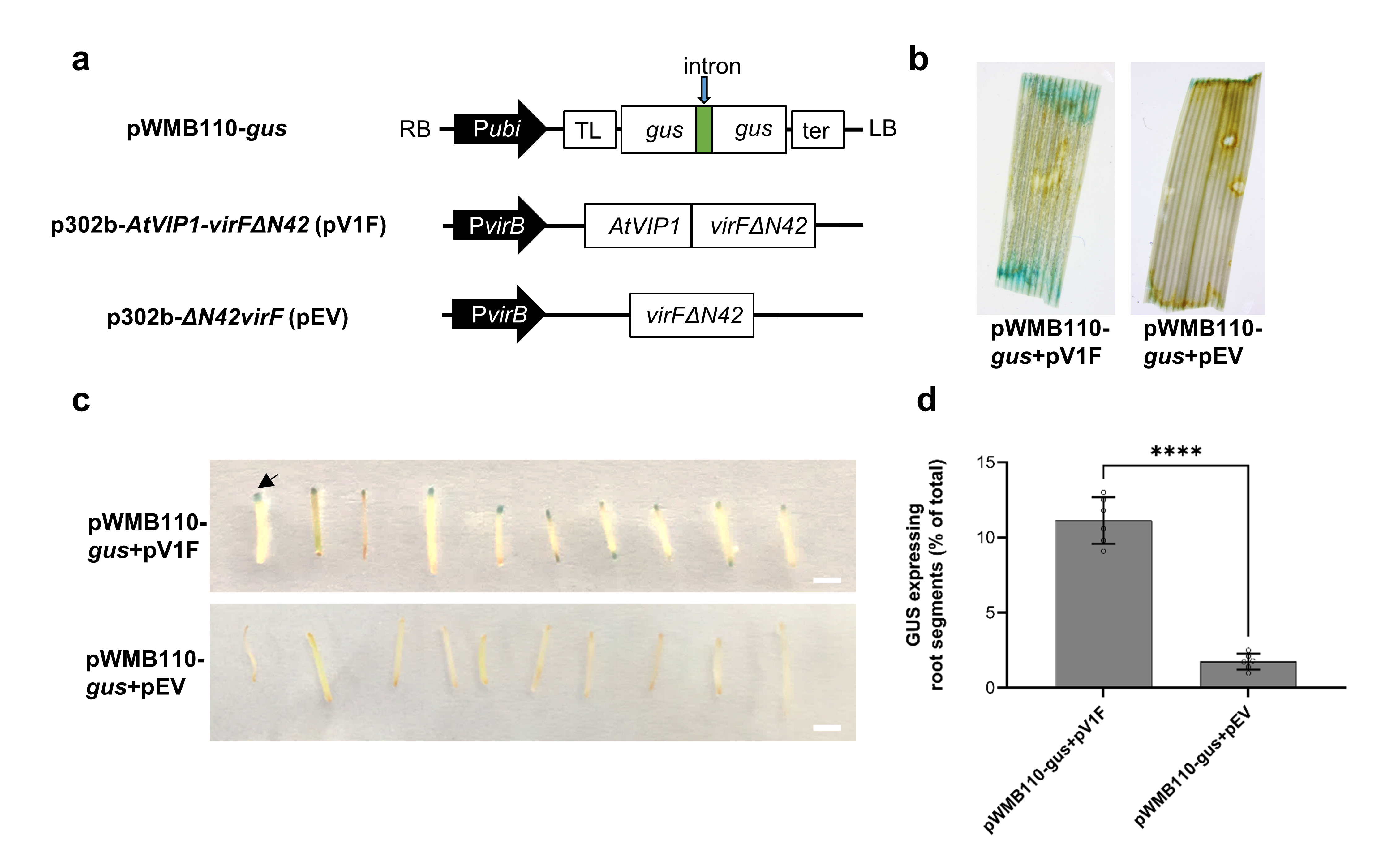

Supplement: S2 Fig — (a) Schematic representation of plasmid constructs used in this experiment. The pWMB110-gus construct contains a β-glucuronidase expression cassette, carrying the maize adh1 intron, in the T-DNA region. (b-c) Transient transformation on wheat leaf and root segments. Dissected wheat tissue segments were inoculated with A. tumefaciens EHA105 carrying the binary plasmid pWMB110-gus and either pV1F or the control plasmid pEV. At 3 days post inoculation, GUS activity was analyzed by histochemical staining. Scale bars = 2 mm. At least 50 leaf or root segments were recorded in each experiment. Each experiment was repeated 3 times and representative results were presented. (d) Quantification of root segments that expressed the gus gene in (c). Statistical differences among the samples were analyzed with Šídák’s multiple comparisons test (P< 0.0001: ****). (TIF) [file ppat.1011346.s006.tif]

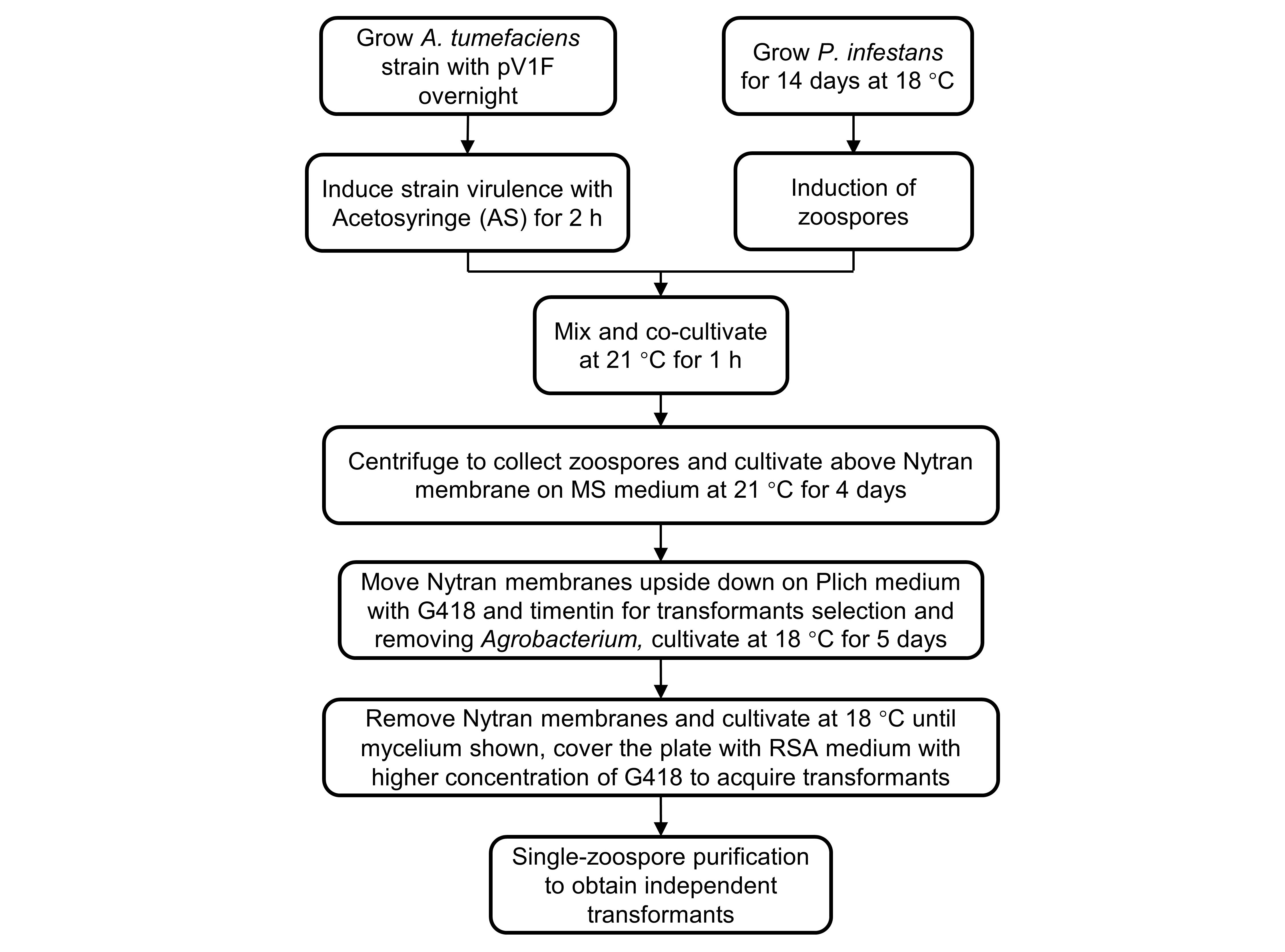

Supplement: S3 Fig — (TIF) [file ppat.1011346.s007.tif]

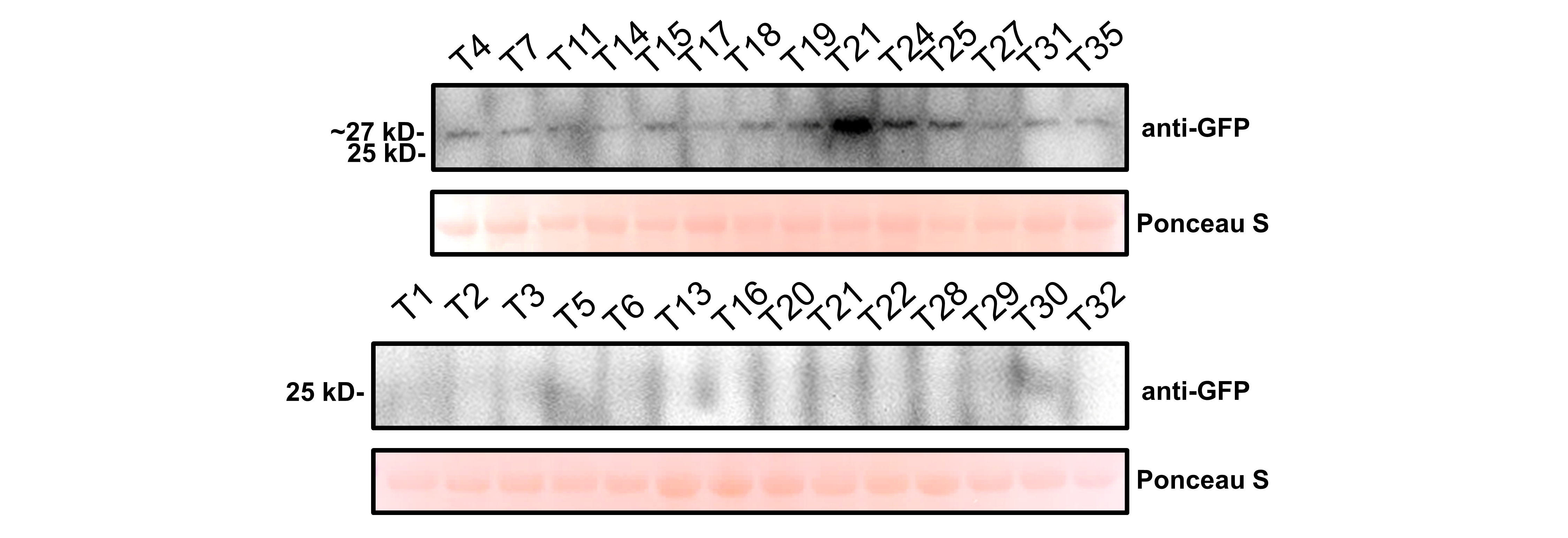

Supplement: S4 Fig — Total protein samples were purified from 14 transformants with a GFP signal (up) and 14 transformants without a GFP signal (down). All blots were probed with an anti-GFP antibody. The protein blot was stained with Ponceau S to confirm equal loading. (TIF) [file ppat.1011346.s008.tif]

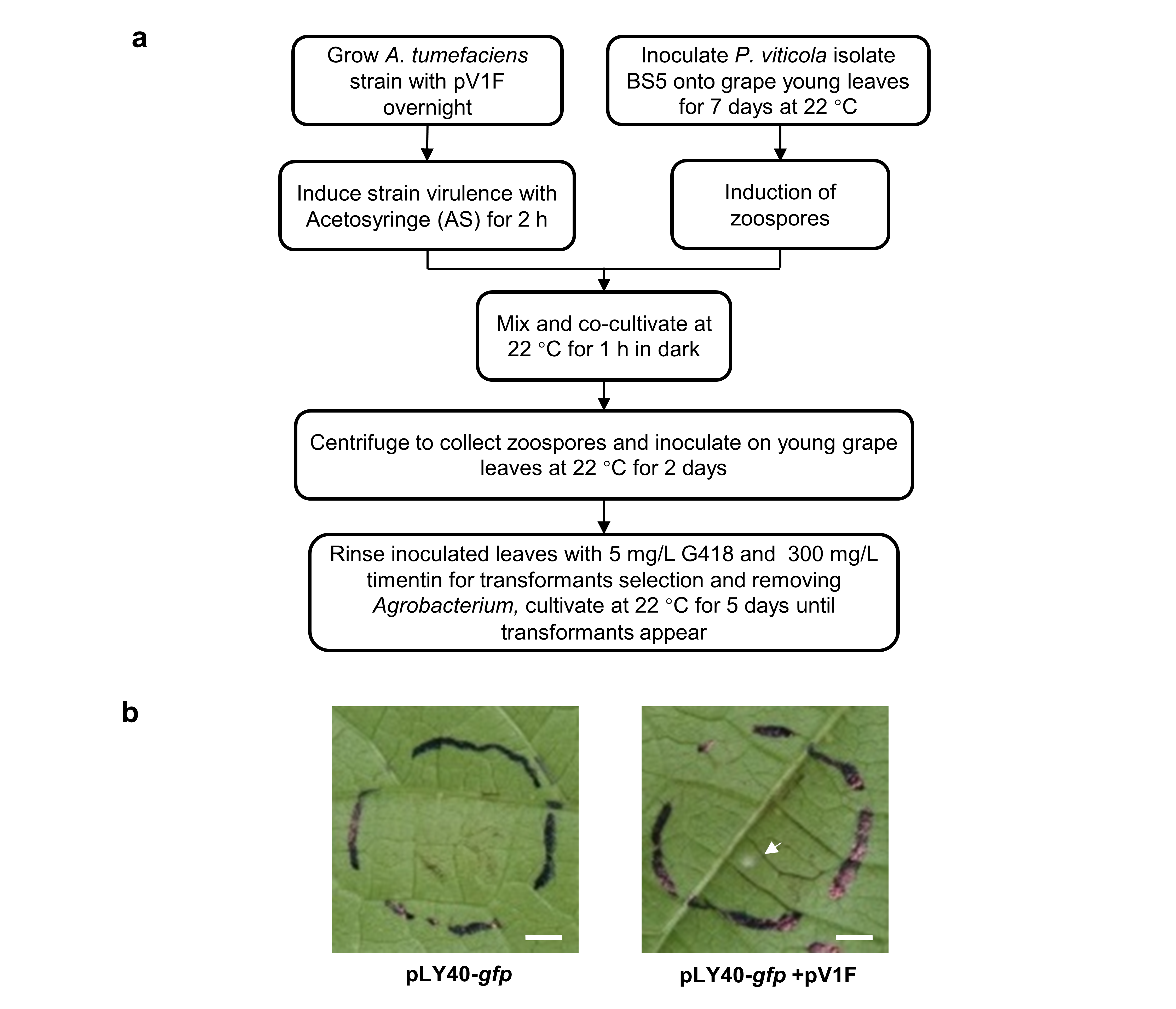

Supplement: S5 Fig — (a) Schematic outline of the optimized AMT method for P. viticola BS5. (b) AMT with only pLY40-gfp produced no G418 resistant transformants of P. viticola BS5 (left), while AMT with pLY40-gfp and pV1F produced the transformant T1 (white arrow) that is resistant to G418 (right). Scale bars = 2 mm. (TIF) [file ppat.1011346.s009.tif]

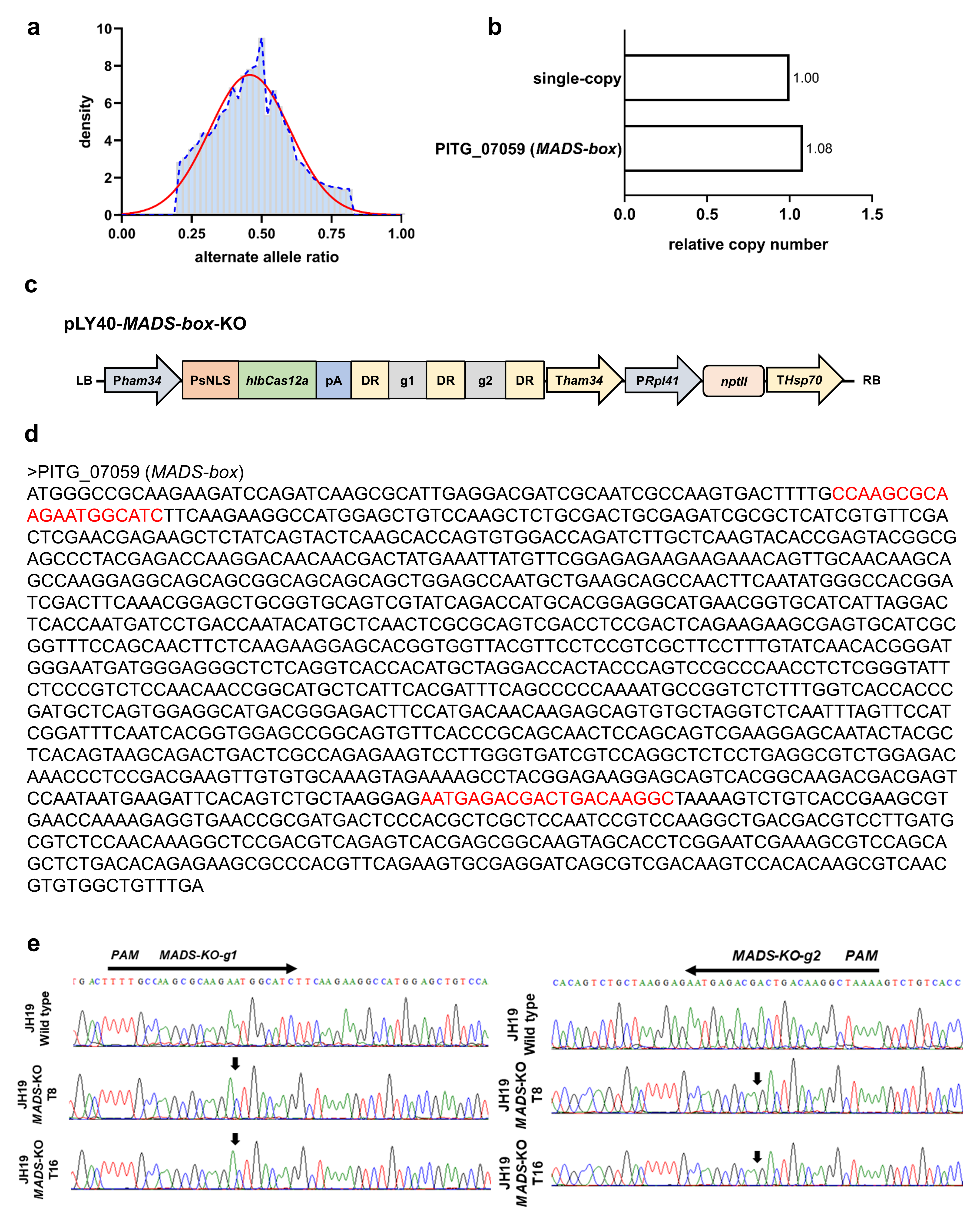

Supplement: S6 Fig — (a) Genome-wide allele ratio analysis of P. infestans JH19. (b) Copy number of PITG_07059 (MADS-box) relative to single-copy control gene (= 1.0), determined based on read depth in DNA library of JH19 strain. (c) Schematic representation of the constructs used in this experiment. The pLY40-MADS-box-KO with either pV1F or pEV were used for P. infestans transformation in this experiment. Two gRNAs for MADS-box editing are named as g1 and g2. (d) Gene sequence of PITG_07059 (MADS-box). Sequences marked in red are targeted by g1 and g2. (e) Sequencing chromatograms of MADS-box in wild type JH19, T8 and T16. Both T8 and T16 showed single peaks in either the g1 (left) or g2 (right) target sites. The wild type sequences with gRNA targets are shown at the top of the panel; black arrows indicate the 5’ border of the detected deletion. (TIF) [file ppat.1011346.s010.tif]

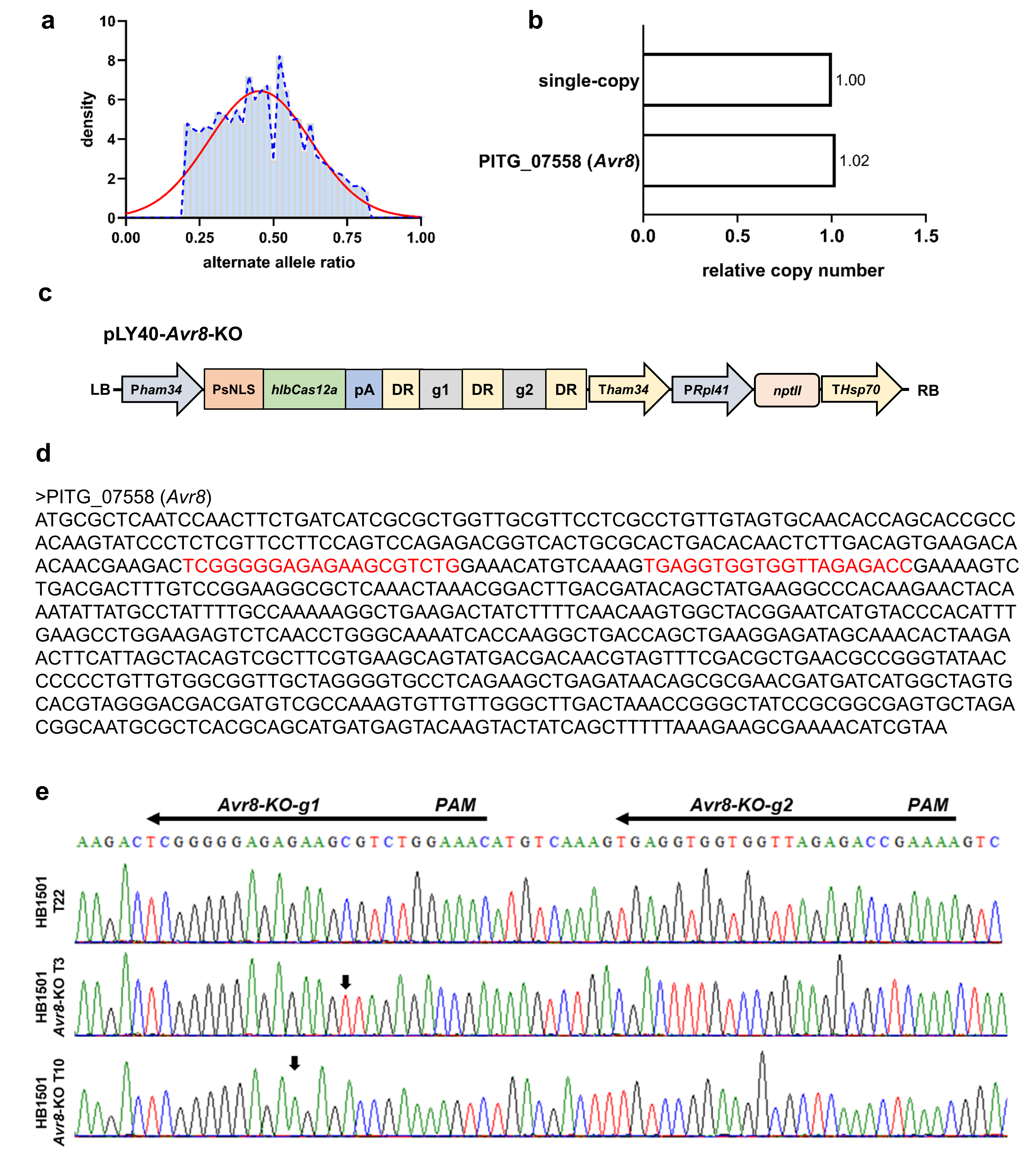

Supplement: S7 Fig — (a) Genome-wide allele ratio analysis of P. infestans HB1501. (b) Copy number of PITG_07558 (Avr8) relative to single-copy control gene (= 1.0), determined based on read depth in DNA library of HB1501 strain. (c) Schematic representation of the constructs used in this experiment. (d) Gene sequence of PITG_07558 (Avr8). Two selected gRNA target regions are marked in red. (e) Sequencing chromatograms of Avr8 in T3, T10 and T22 of HB1501. Both T3 and T22 showed single peaks in both g1 and g2 target sites. The wild type sequences with gRNA targets are shown at the top of the panel; black arrows indicate the 5’ border of the detected deletion. (TIF) [file ppat.1011346.s011.tif]

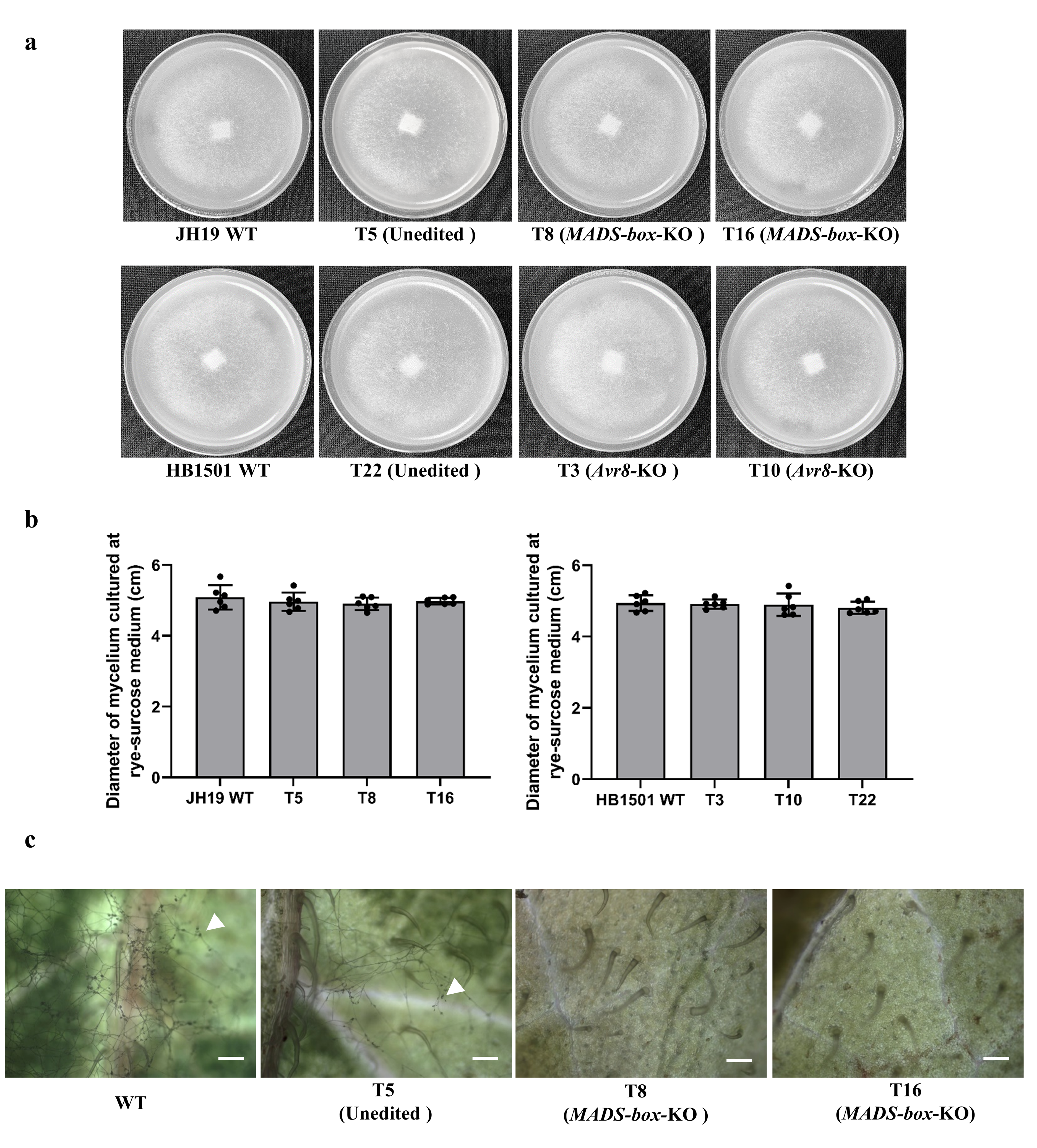

Supplement: S8 Fig — (a) Mycelia cultured on the rye-sucrose medium were photographed at 5 days post inoculation. (b) Quantification of mycelium diameter of P. infestans strains in (a). All data represent average values from three independent experiments with the indicated standard deviations. (c) Microscopy images of the opposite side of the inoculated region of detached potato leaves in Fig 5E show the details of mycelia generated during infection. Images were taken at 5 days post inoculation. White arrowheads indicate the observed sporangia. Scale bars = 1 mm. (TIF) [file ppat.1011346.s012.tif]
