# Supplementary material for: A modified Agrobacterium-mediated transformation for two oomycete pathogens
Source: PLoS Pathog. 2023 Apr 21;19(4):e1011346. doi: 10.1371/journal.ppat.1011346 (PMC10156060; doi:10.1371/journal.ppat.1011346)
Supplement: S1 Appendix — (DOCX) [file ppat.1011346.s013.docx]

Sequences of pLY40, pLY40-gfp, pLY40-Cas12a-gfp

*all HindIII restriction sites were highlighted

>pLY40

TGCGCGTGCCTTTGATCGCCCGCGACACGACAAAGGCCGCTTGTAGCCTTCCATCCGTGACCTCAATGCGCTGCTTAACCAGCTCCACCAGGTCGGCGGTGGCCCATATGTCGTAAGGGCTTGGCTGCACCGGAATCAGCACGAAGTCGGCTGCCTTGATCGCGGACACAGCCAAGTCCGCCGCCTGGGGCGCTCCGTCGATCACTACGAAGTCGCGCCGGCCGATGGCCTTCACGTCGCGGTCAATCGTCGGGCGGTCGATGCCGACAACGGTTAGCGGTTGATCTTCCCGCACGGCCGCCCAATCGCGGGCACTGCCCTGGGGATCGGAATCGACTAACAGAACATCGGCCCCGGCGAGTTGCAGGGCGCGGGCTAGATGGGTTGCGATGGTCGTCTTGCCTGACCCGCCTTTCTGGTTAAGTACAGCGATAACCTTCATGCGTTCCCCTTGCGTATTTGTTTATTTACTCATCGCATCATATACGCAGCGACCGCATGACGCAAGCTGTTTTACTCAAATACACATCACCTTTTTAGACGGCGGCGCTCGGTTTCTTCAGCGGCCAAGCTGGCCGGCCAGGCCGCCAGCTTGGCATCAGACAAACCGGCCAGGATTTCATGCAGCCGCACGGTTGAGACGTGCGCGGGCGGCTCGAACACGTACCCGGCCGCGATCATCTCCGCCTCGATCTCTTCGGTAATGAAAAACGGTTCGTCCTGGCCGTCCTGGTGCGGTTTCATGCTTGTTCCTCTTGGCGTTCATTCTCGGCGGCCGCCAGGGCGTCGGCCTCGGTCAATGCGTCCTCACGGAAGGCACCGCGCCGCCTGGCCTCGGTGGGCGTCACTTCCTCGCTGCGCTCAAGTGCGCGGTACAGGGTCGAGCGATGCACGCCAAGCAGTGCAGCCGCCTCTTTCACGGTGCGGCCTTCCTGGTCGATCAGCTCGCGGGCGTGCGCGATCTGTGCCGGGGTGAGGGTAGGGCGGGGGCCAAACTTCACGCCTCGGGCCTTGGCGGCCTCGCGCCCGCTCCGGGTGCGGTCGATGATTAGGGAACGCTCGAACTCGGCAATGCCGGCGAACACGGTCAACACCATGCGGCCGGCCGGCGTGGTGGTGTCGGCCCACGGCTCTGCCAGGCTACGCAGGCCCGCGCCGGCCTCCTGGATGCGCTCGGCAATGTCCAGTAGGTCGCGGGTGCTGCGGGCCAGGCGGTCTAGCCTGGTCACTGTCACAACGTCGCCAGGGCGTAGGTGGTCAAGCATCCTGGCCAGCTCCGGGCGGTCGCGCCTGGTGCCGGTGATCTTCTCGGAAAACAGCTTGGTGCAGCCGGCCGCGTGCAGTTCGGCCCGTTGGTTGGTCAAGTCCTGGTCGTCGGTGCTGACGCGGGCATAGCCCAGCAGGCCAGCGGCGGCGCTCTTGTTCATGGCGTAATGTCTCCGGTTCTAGTCGCAAGTATTCTACTTTATGCGACTAAAACACGCGACAAGAAAACGCCAGGAAAAGGGCAGGGCGGCAGCCTGTCGCGTAACTTAGGACTTGTGCGACATGTCGTTTTCAGAAGACGGCTGCACTGAACGTCAGAAGCCGACTGCACTATAGCAGCGGAGGGGTTGGATCAAAGTACTTTGATCCCGAGGGGAACCCTGTGGTTGGCATGCACATACAAATGGACGAACGGATAAACCTTTTCACGCCCTTTTAAATATCCGTTATTCTAATAAACGCTCTTTTCTCTTAGGTTTACCCGCCAATATATCCTGTCAAACACTGATAGTTTAAACTGAAGGCGGGAAACGACAATCTGATCCAAGCTCAAGCTAAGCTTCAGGAAACAGCTATGACCATGTAATACGACTCACTATAGGGGATATCGCGGCCGCGTTAACGCTACCATGGAGCTCCAAATAATGATTTTATTTTGACTGATAGTGACCTGTTCGTTGCAACAAATTGATAAGCAATGCTTTTTTATAATGCCAACTTTGTATAATAAAGTTGAACGAGAAACGTAAAATGATATAAATATCAATATATTAAATTAGATTTTGCATAAAAAACAGACTACATAATACTGTAAAACACAACATATGCAGTCACTATGAATCAACTACTTAGATGGTATTAGTGACCTGTAGAATTCGAGCTCGACCGACAGCCTTCCAAATGTTCTTCTCAAACGGAATCGTCGTATCCAGCCTACTCGCTATTGTCCTCAATGCCGTATTAAATCATAAAAAGAAATAAGAAAAAGAGGTGCGAGCCTCTTTTTTGTGTGACAAAATAAAAACATCTACCTATTCATATACGCTAGTGTCATAGTCCTGAAAATCATCTGCATCAAGAACAATTTCACAACTCTTATACTTTTCTCTTACAAGTCGTTCGGCTTCATCTGGATTTTCAGCCTCTATACTTACTAAACGTGATAAAGTTTCTGTAATTTCTACTGTATCGACCTGCAGACTGGCTGTGTATAAGGGAGCCTGACATTTATATTCCCCAGAACATCAGGTTAATGGCGTTTTTGATGTCATTTTCGCGGTGGCTGAGATCAGCCACTTCTTCCCCGATAACGGAGACCGGCACACTGGCCATATCGGTGGTCATCATGCGCCAGCTTTCATCCCCGATATGCACCACCGGGTAAAGTTCACGGGAGACTTTATCTGACAGCAGACGTGCACTGGCCAGGGGGATCACCATCCGTCGCCCGGGCGTGTCAATAATATCACTCTGTACATCCACAAACAGACGATAACGGCTCTCTCTTTTATAGGTGTAAACCTTAAACTGCATTTCACCAGTCCCTGTTCTCGTCAGCAAAAGAGCCGTTCATTTCAATAAACCGGGCGACCTCAGCCATCCCTTCCTGATTTTCCGCTTTCCAGCGTTCGGCACGCAGACGACGGGCTTCATTCTGCATGGTTGTGCTTACCAGACCGGAGATATTGACATCATATATGCCTTGAGCAACTGATAGCTGTCGCTGTCAACTGTCACTGTAATACGCTGCTTCATAGCACACCTCTTTTTGACATACTTCGGGTATACATATCAGTATATATTCTTATACCGCAAAAATCAGCGCGCAAATACGCATACTGTTATCTGGCTTTTAGTAAGCCGGATCCACGCGATTACGCCCCGCCCTGCCACTCATCGCAGTACTGTTGTAATTCATTAAGCATTCTGCCGACATGGAAGCCATCACAGACGGCATGATGAACCTGAATCGCCAGCGGCATCAGCACCTTGTCGCCTTGCGTATAATATTTGCCCATGGTGAAAACGGGGGCGAAGAAGTTGTCCATATTGGCCACGTTTAAATCAAAACTGGTGAAACTCACCCAGGGATTGGCTGAGACGAAAAACATATTCTCAATAAACCCTTTAGGGAAATAGGCCAGGTTTTCACCGTAACACGCCACATCTTGCGAATATATGTGTAGAAACTGCCGGAAATCGTCGTGGTATTCACTCCAGAGCGATGAAAACGTTTCAGTTTGCTCATGGAAAACGGTGTAACAAGGGTGAACACTATCCCATATCACCAGCTCACCGTCTTTCATTGCCATACGGAATTCCGGATGAGCATTCATCAGGCGGGCAAGAATGTGAATAAAGGCCGGATAAAACTTGTGCTTATTTTTCTTTACGGTCTTTAAAAAGGCCGTAATATCCAGCTGAACGGTCTGGTTATAGGTACATTGAGCAACTGACTGAAATGCCTCAAAATGTTCTTTACGATGCCATTGGGATATATCAACGGTGGTATATCCAGTGATTTTTTTCTCCATTTTAGCTTCCTTAGCTCCTGAAAATCTCGATAACTCAAAAAATACGCCCGGTAGTGATCTTATTTCATTATGGTGAAAGTTGGAACCTCTTACGTGCCGATCAACGTCTCATTTTCGCCAAAAGTTGGCCCAGGGCTTCCCGGTATCAACAGGGACACCAGGATTTATTTATTCTGCGAAGTGATCTTCCGTCACAGGTATTTATTCGGCGCAAAGTGCGTCGGGTGATGCTGCCAACTTAGTCGACGCTCTAGAGCTCCAAATAATGATTTTATTTTGACTGATAGTGACCTGTTCGTTGCAACAAATTGATAAGCAATGCTTTTTTATAATGCCAACTTTGTACAAGAAAGTTGAACGAGAAACGTAAAATGATATAAATATCAATATATTAAATTAGATTTTGCATAAAAAACAGACTACATAATACTGTAAAACACAACATATGCAGTCACTATGAATCAACTACTTAGATGGTATTAGTGACCTGTAGAATTCGAGCTCTAGAGCTGCAGGGCCCGAGCTTAAGACTGGCCGTCGTTTTACGGTACCGCGATCGCTCGCGACCTGCAGGCATAAAGCCGTCAGTGTCCGCATAAAGAACCACCCATAATACCCATAATAGCTGTTTGCCATCGCTACCTTAGTTATACCTCGTATTCGATTAAATGATACAACTCATTGCCACACCGCAAGACGGCGTCACCACCATAGACAGCCTTTCAAGCACCCCCTTGTCTATGAGCTGCCACGTCAGCTGCCGCTCCTAATATCCACCCGCGTCCCTATTCCCGTCATATGGACGTACTTGATGGTAAAAATCAAGGGCCAACTGACTACCCACGGAAAAATCGCTATCCCATCAAAGCAGTCGAGTTTTTCCTTTTTTTTTCTTATCTTTTTCAAGTCAAATCAAAATGTCGCTACCATAACCATTATACCGCCCTCAAAATAAAGTCTATCCCTCCTATCCACTGGTCGGTTCCTCTCCCTCTGATGGACAAAGGGTCGCCTCTACGTACCCGCGACAATCTCGGCTTTTCCAATGTCGACAGCTGGCATATCGCACGCGAGGTTAGGCGTCTTCAGCCGGCGTAACCACAAACCTCGAGTCAAGTACCCTTTTTCAGACCTTAATTTCCGTGTCCATTCCCTCGCGTTTACACGAAGGTTTCTGAGAAATACCACATGTCACAGAGAGAGAGAAGATTGGAGAACCTTCTCAACCTGACCCGTGCAGAGCGCTTTTGCGTCCTACCATCCGTTAATTATAGCACCACCACCACATGTCTCGCCGGAACTCTCTTATCCGCTCTTTTTGATCGTCTTTTTTGCGCCCCATTAAAGACATCTCTTGCACAGGGATCGTTTCTGATTGGCCATTTCACTTCCTCTTTTACATCCGAAAACCACCTGTTTGTGGGGTTGTGAAGCCACAACCGGACGCTCAAAAAGCCTCGCCCGACTCGCCCACGATCGGAAGGCTCATTCTCCTTTTCACTCTCACGTGCCCAAGTCCCAACCGACTCTTTTTCGACCTTCACTCTCACCGACAACCCGGCTCTAACTATAACGGTCCTAAGGTAGCGACCTTGACGTCTAACCGCGGGGTGCCTACTTCTTGTAAGCCGAAGGTCGAGCTGTGTGCACTGAGTGCGAAGTTGCAGATTTTGTGTCTATGCATTTAATACAATTTCCTTTTTTCCATTTTCGTTGTTTTTTTTTGTGAAAGAACGATTGTTTTCAAAAGAAGGCCGTTTGTAGAACAATTTTTTTTTATACTAGCATTTGGTATTCGAGTGTGACGAATCGGTGATGGCGCTTTACAAATGAAATATAACGCTGAACAAGCGCAGTGACAGCTTTATTTTATAACAGAGAATAAAGACCAAAGTAGAGTAGACTGATGGCGGCTTGAAACAAAACTACGTGACTTCTAATGCAAAGAAAACCATACCCCACGCTACTTAGAATTGTTTCTCTTTGGTTGATGACCGCTATAGTCGTATGGAGTGATCCCTTTTTGTCAATATTTCGATTCTTGCTAAAACCGTGACTATTTTGAGGCAACTGTGTGTACTTAACATGGTAGAAATTTCTATGTTTAAGCCAGCTGACAATCATTCTTTATTTATCGTAAACCAACCATTAGGACCGTTATAGTTAATTACCCTGTTATCCCTATTAATTAAGAGCTCGCTACCTTAAGAGAGGATATCGGCGCGTCCGTCATTTCCTCGCAGCAACTCGCTTCCACGATTTCTTTGTATTCACTCGGCAGCCTCGAGCTGATTGAGCGAGAGATGGACGTCCACGCCGATGGATCACCCGTTTGGCCGCAATATTTGCCTGGACTGTTAGATCCCGTACCAGAGATAGACATGGTCAGCGACGGCTTCATTACCTCGTCGGAATCATTGCTGGAACATCAAACAAGTTTTATCTACGGCTTCGATAACCCTGAGTCGACGGCATCATCTGGAGCATTGGTAGATGCTGATGACGTGTGCTCTTCCACCACTTCTAGACTGTGTATCAAGCTCCGGTTCACAGGCGCCGATCAGTTGGCTCCAATATTTCTTCGTAAACTGCAGAGCATTCACGCTGCACTATCTTCGTCAATTGGTCTTCCTCCCGGATCAAGTGTTGCTGGTTCAACTTCTACTTATCCGGCTCCGTGGGTGCTGGTTGGTGCTCAGAGTCTCGGTGGGGATACTTCGTGGTCCTTCTTCGTAGGCGGGTCGTCATCAAGCGGCAATGCCCAGAGCTTCCGTCTCACGTTCGAAGCTGTAAACTCGATGCAAGATATGACCACTACCACACCCTTCACCATAGACCTGGAGTTTGTTCCTGACGACCCAACTGTCATCCCCATTGTGCGACGTCGGCAGTTTGGCGACCTCGCGGTGGTAGACACCACTGGCCAGCCTGAACCAGCATTTATCACCTCTGAGATTCCTGATCTGATCGACGATGGAGACGAAGTCACTGGTATCACCATAACATTGGATTTGGAGCAGCCTCGACTTGGTGGACTGTATTCAGGTTCACAACTGGATGATGTAGCCCCAACTTGCAGCGAGTGCACGACGTTATTGGAGGATTGCAGCAGCTCGCCCGAGTGCCGAGCGTTCTCAGCGTGTATGGGTGTTCTGCTTGAGGCAGACTTGACACTGATAGCTACGATGCTGGAGAAGGACGATATCGACAGCAGCGTTGATGCAACTTGGCTGCTGCAGGACTGCTTGAGTCCATCGGACGGCACGGAATGGTCGAGTACAGTGCGTGAGCTGCTCGAGTCCAGCTACGCTTGTCTGTGGGCGAAGAGCTGTCCACTGGCATACAGCACCACAATAGGGAGGCAGATCGTGCTGGACTACAGTCCTGGAGAGCAGATCTTGACCTTCGACCTGGCGAGCGGATTCGCCAGCGTCAGCTTCGAGCTCGGCAACTGGGCGTACAGTTTTGTCGAGGACTTCACGGCGAACGCGACGGAAGTGACAGCCCAACTGGACACGATGCTGATGGAGATGTACCGCACTGCGCTGTCGAATGTGGCGACGGTGAAGTCGGCCCTCGTCACGACGGAAGACTCGAACACGGGAGTGGTGACGGCGCAGTTGACCATTCTCTACGAGTTCCTGGGCGCACTGCCACAGCCTTATGTCGCAGAGAATGGAGGTTCGTTGGCGTGCAATACCACCGAGGAGGCTCTGCATCTGCGCGTACTAGCCATAACGTAAGAACGAGTGCAATAACAAGAACAACTTCACTTGCAGTGCAAGTTGAAGACACTATAGTGGGGAAAGTGTCACTCAAGTGGGGTCACATTTTGGTTGGCCAGTAGTCCGCGCCCGCTGTCCAGCCAATCAAAGTGTTTGAAGATTCCATCTCCTCTTTTTTGCACCATACAACGCCCTTCATGCGGGACTTGTTGCCTACAATAACCAAGTCTCTCCACGCTGGCAAGCTCGCAGCCAACTATCCTCTCGCATGTTGATCCCAAGGCTGCGCTCGCCGACAGCAACAAGCCTCACTTTCTGCTGACTGCTCTGCTTGGAACATCGTTCGGGGTAAGTGGCGACCCCCTTGTCTCTCTCTGAGCCTCGCAGCTATTGGCCCCACATGGCTACATCGACAGTCTTTACTTACTATAGTTTTAATTCGTTCGGTGACGCTAGCATCTGAGCATCCACCCGGGCAACAATGATTGAACAAGATGGATTGCACGCAGGTTCTCCGGCCGCTTGGGTGGAGAGGCTATTCGGCTATGACTGGGCACAACAGACAATCGGCTGCTCTGATGCCGCCGTGTTCCGGCTGTCAGCGCAGGGGCGCCCGGTTCTTTTTGTCAAGACCGACCTGTCCGGTGCCCTGAATGAACTGCAGGACGAGGCAGCGCGGCTATCGTGGCTGGCCACGACGGGCGTTCCTTGCGCAGCTGTGCTCGACGTTGTCACTGAAGCGGGAAGGGACTGGCTGCTATTGGGCGAAGTGCCGGGGCAGGATCTCCTGTCATCTCACCTTGCTCCTGCCGAGAAAGTATCCATCATGGCTGATGCAATGCGGCGGCTGCATACGCTTGATCCGGCTACCTGCCCATTCGACCACCAAGCGAAACATCGCATCGAGCGAGCACGTACTCGGATGGAAGCCGGTCTTGTCGATCAGGATGATCTGGACGAAGAGCATCAGGGGCTCGCGCCAGCCGAACTGTTCGCCAGGCTCAAGGCGCGCATGCCCGACGGCGAGGATCTCGTCGTGACCCATGGCGATGCCTGCTTGCCGAATATCATGGTGGAAAATGGCCGCTTTTCTGGATTCATCGACTGTGGCCGGCTGGGTGTGGCGGACCGCTATCAGGACATAGCGTTGGCTACCCGTGATATTGCTGAAGAGCTTGGCGGCGAATGGGCTGACCGCTTCCTCGTGCTTTACGGTATCGCCGCTCCCGATTCGCAGCGCATCGCCTTCTATCGCCTTCTTGACGAGTTCTTCTGATAGGTACCGGTGTCGACTAGAATACTTAGCAATTGGTTAGTTTGAAAATTGTCAAAGCAGAGTTTTTTCGACAATAAAATTTATTTTCTAAATATTTGATTTAGCGTTGACTTAATTTGTTGATTCGGTTGTCATCTTTTGTGAAGATGCCCGAGAGTCTAGTAAATCTCAAGCGAAACATAGTGAGAAGGTCACTATGGCTGGTTTGCATGAGTCTGGGCCTATTGTGCTTACGCCGCCTTAGTAACAAACATGCCAAAAGAAGGAAAGCTTTCACCAGCTTTCAAGTTTAATCAATCTTTGAGAGTCACAATTGCAAGAATAAATGAATTTTTACAACACAGTTCGATGCACTGTGTTTCGTCTTGATGAAAATTGGACGGCAAATCGAGTATCTTAAACAAATCTCGGCATAGGCTGTCCATTTTGTGGTCGCCTCTTTGTTGGTAGTTTGCAGTAGTTTCATTAGTAGAAGATCTGCATCCTCAATCTGCTAAACGTCTTGAGCAGCTATGAGACGATCCGGGGAATTGGGGCGCGCCTCTAGAATTTAAATGGATCCTACGTACTCGAGGAATTCAATTCGGCGTTAATTCAGTACATTAAAAACGTCCGCAATGTGTTATTAAGTTGTCTAAGCGTCAATTTGTTTACACCACAATATATCCTGCCACCAGCCAGCCAACAGCTCCCCGACCGGCAGCTCGGCACAAAATCACCACTCGATACAGGCAGCCCATCAGTCCGGGACGGCGTCAGCGGGAGAGCCGTTGTAAGGCGGCAGACTTTGCTCATGTTACCGATGCTATTCGGAAGAACGGCAACTAAGCTGCCGGGTTTGAAACACGGATGATCTCGCGGAGGGTAGCATGTTGATTGTAACGATGACAGAGCGTTGCTGCCTGTGATCAATTCGGGCACGAACCCAGTGGACATAAGCCTCGTTCGGTTCGTAAGCTGTAATGCAAGTAGCGTAACTGCCGTCACGCAACTGGTCCAGAACCTTGACCGAACGCAGCGGTGGTAACGGCGCAGTGGCGGTTTTCATGGCTTCTTGTTATGACATGTTTTTTTGGGGTACAGTCTATGCCTCGGGCATCCAAGCAGCAAGCGCGTTACGCCGTGGGTCGATGTTTGATGTTATGGAGCAGCAACGATGTTACGCAGCAGGGCAGTCGCCCTAAAACAAAGTTAAACATCATGGGGGAAGCGGTGATCGCCGAAGTATCGACTCAACTATCAGAGGTAGTTGGCGTCATCGAGCGCCATCTCGAACCGACGTTGCTGGCCGTACATTTGTACGGCTCCGCAGTGGATGGCGGCCTGAAGCCACACAGTGATATTGATTTGCTGGTTACGGTGACCGTAAGGCTTGATGAAACAACGCGGCGAGCTTTGATCAACGACCTTTTGGAAACTTCGGCTTCCCCTGGAGAGAGCGAGATTCTCCGCGCTGTAGAAGTCACCATTGTTGTGCACGACGACATCATTCCGTGGCGTTATCCAGCTAAGCGCGAACTGCAATTTGGAGAATGGCAGCGCAATGACATTCTTGCAGGTATCTTCGAGCCAGCCACGATCGACATTGATCTGGCTATCTTGCTGACAAAAGCAAGAGAACATAGCGTTGCCTTGGTAGGTCCAGCGGCGGAGGAACTCTTTGATCCGGTTCCTGAACAGGATCTATTTGAGGCGCTAAATGAAACCTTAACGCTATGGAACTCGCCGCCCGACTGGGCTGGCGATGAGCGAAATGTAGTGCTTACGTTGTCCCGCATTTGGTACAGCGCAGTAACCGGCAAAATCGCGCCGAAGGATGTCGCTGCCGACTGGGCAATGGAGCGCCTGCCGGCCCAGTATCAGCCCGTCATACTTGAAGCTAGACAGGCTTATCTTGGACAAGAAGAAGATCGCTTGGCCTCGCGCGCAGATCAGTTGGAAGAATTTGTCCACTACGTGAAAGGCGAGATCACCAAGGTAGTCGGCAAATAATGTCTAGCTAGAAATTCGTTCAAGCCGACGCCGCTTCGCCGGCGTTAACTCAAGCGATTAGATGCACTAAGCACATAATTGCTCACAGCCAAACTATCAGGTCAAGTCTGCTTTTATTATTTTTAAGCGTGCATAATAAGCCCTACACAAATTGGGAGATATATCATGCATGACCAAAATCCCTTAACGTGAGTTTTCGTTCCACTGAGCGTCAGACCCCGTAGAAAAGATCAAAGGATCTTCTTGAGATCCTTTTTTTCTGCGCGTAATCTGCTGCTTGCAAACAAAAAAACCACCGCTACCAGCGGTGGTTTGTTTGCCGGATCAAGAGCTACCAACTCTTTTTCCGAAGGTAACTGGCTTCAGCAGAGCGCAGATACCAAATACTGTCCTTCTAGTGTAGCCGTAGTTAGGCCACCACTTCAAGAACTCTGTAGCACCGCCTACATACCTCGCTCTGCTAATCCTGTTACCAGTGGCTGCTGCCAGTGGCGATAAGTCGTGTCTTACCGGGTTGGACTCAAGACGATAGTTACCGGATAAGGCGCAGCGGTCGGGCTGAACGGGGGGTTCGTGCACACAGCCCAGCTTGGAGCGAACGACCTACACCGAACTGAGATACCTACAGCGTGAGCTATGAGAAAGCGCCACGCTTCCCGAAGGGAGAAAGGCGGACAGGTATCCGGTAAGCGGCAGGGTCGGAACAGGAGAGCGCACGAGGGAGCTTCCAGGGGGAAACGCCTGGTATCTTTATAGTCCTGTCGGGTTTCGCCACCTCTGACTTGAGCGTCGATTTTTGTGATGCTCGTCAGGGGGGCGGAGCCTATGGAAAAACGCCAGCAACGCGGCCTTTTTACGGTTCCTGGCCTTTTGCTGGCCTTTTGCTCACATGTTCTTTCCTGCGTTATCCCCTGATTCTGTGGATAACCGTATTACCGCCTTTGAGTGAGCTGATACCGCTCGCCGCAGCCGAACGACCGAGCGCAGCGAGTCAGTGAGCGAGGAAGCGGAAGAGCGCCTGATGCGGTATTTTCTCCTTACGCATCTGTGCGGTATTTCACACCGCATATGGTGCACTCTCAGTACAATCTGCTCTGATGCCGCATAGTTAAGCCAGTATACACTCCGCTATCGCTACGTGACTGGGTCATGGCTGCGCCCCGACACCCGCCAACACCCGCTGACGCGCCCTGACGGGCTTGTCTGCTCCCGGCATCCGCTTACAGACAAGCTGTGACCGTCTCCGGGAGCTGCATGTGTCAGAGGTTTTCACCGTCATCACCGAAACGCGCGAGGCAGGGTGCCTTGATGTGGGCGCCGGCGGTCGAGTGGCGACGGCGCGGCTTGTCCGCGCCCTGGTAGATTGCCTGGCCGTAGGCCAGCCATTTTTGAGCGGCCAGCGGCCGCGATAGGCCGACGCGAAGCGGCGGGGCGTAGGGAGCGCAGCGACCGAAGGGTAGGCGCTTTTTGCAGCTCTTCGGCTGTGCGCTGGCCAGACAGTTATGCACAGGCCAGGCGGGTTTTAAGAGTTTTAATAAGTTTTAAAGAGTTTTAGGCGGAAAAATCGCCTTTTTTCTCTTTTATATCAGTCACTTACATGTGTGACCGGTTCCCAATGTACGGCTTTGGGTTCCCAATGTACGGGTTCCGGTTCCCAATGTACGGCTTTGGGTTCCCAATGTACGTGCTATCCACAGGAAAGAGACCTTTTCGACCTTTTTCCCCTGCTAGGGCAATTTGCCCTAGCATCTGCTCCGTACATTAGGAACCGGCGGATGCTTCGCCCTCGATCAGGTTGCGGTAGCGCATGACTAGGATCGGGCCAGCCTGCCCCGCCTCCTCCTTCAAATCGTACTCCGGCAGGTCATTTGACCCGATCAGCTTGCGCACGGTGAAACAGAACTTCTTGAACTCTCCGGCGCTGCCACTGCGTTCGTAGATCGTCTTGAACAACCATCTGGCTTCTGCCTTGCCTGCGGCGCGGCGTGCCAGGCGGTAGAGAAAACGGCCGATGCCGGGATCGATCAAAAAGTAATCGGGGTGAACCGTCAGCACGTCCGGGTTCTTGCCTTCTGTGATCTCGCGGTACATCCAATCAGCTAGCTCGATCTCGATGTACTCCGGCCGCCCGGTTTCGCTCTTTACGATCTTGTAGCGGCTAATCAAGGCTTCACCCTCGGATACCGTCACCAGGCGGCCGTTCTTGGCCTTCTTCGTACGCTGCATGGCAACGTGCGTGGTGTTTAACCGAATGCAGGTTTCTACCAGGTCGTCTTTCTGCTTTCCGCCATCGGCTCGCCGGCAGAACTTGAGTACGTCCGCAACGTGTGGACGGAACACGCGGCCGGGCTTGTCTCCCTTCCCTTCCCGGTATCGGTTCATGGATTCGGTTAGATGGGAAACCGCCATCAGTACCAGGTCGTAATCCCACACACTGGCCATGCCGGCCGGCCCTGCGGAAACCTCTACGTGCCCGTCTGGAAGCTCGTAGCGGATCACCTCGCCAGCTCGTCGGTCACGCTTCGACAGACGGAAAACGGCCACGTCCATGATGCTGCGACTATCGCGGGTGCCCACGTCATAGAGCATCGGAACGAAAAAATCTGGTTGCTCGTCGCCCTTGGGCGGCTTCCTAATCGACGGCGCACCGGCTGCCGGCGGTTGCCGGGATTCTTTGCGGATTCGATCAGCGGCCGCTTGCCACGATTCACCGGGGCGTGCTTCTGCCTCGATGCGTTGCCGCTGGGCGGCCTGCGCGGCCTTCAACTTCTCCACCAGGTCATCACCCAGCGCCGCGCCGATTTGTACCGGGCCGGATGGTTTGCGACCGTCACGCCGATTCCTCGGGCTTGGGGGTTCCAGTGCCATTGCAGGGCCGGCAGACAACCCAGCCGCTTACGCCTGGCCAACCGCCCGTTCCTCCACACATGGGGCATTCCACGGCGTCGGTGCCTGGTTGTTCTTGATTTTCCATGCCGCCTCCTTTAGCCGCTAAAATTCATCTACTCATTTATTCATTTGCTCATTTACTCTGGTAGCTGCGCGATGTATTCAGATAGCAGCTCGGTAATGGTCTTGCCTTGGCGTACCGCGTACATCTTCAGCTTGGTGTGATCCTCCGCCGGCAACTGAAAGTTGACCCGCTTCATGGCTGGCGTGTCTGCCAGGCTGGCCAACGTTGCAGCCTTGCTGCTGCGTGCGCTCGGACGGCCGGCACTTAGCGTGTTTGTGCTTTTGCTCATTTTCTCTTTACCTCATTAACTCAAATGAGTTTTGATTTAATTTCAGCGGCCAGCGCCTGGACCTCGCGGGCAGCGTCGCCCTCGGGTTCTGATTCAAGAACGGTTGTGCCGGCGGCGGCAGTGCCTGGGTAGCTCACGCGCTGCGTGATACGGGACTCAAGAATGGGCAGCTCGTACCCGGCCAGCGCCTCGGCAACCTCACCGCCGT

>pLY40-gfp

TGCGCGTGCCTTTGATCGCCCGCGACACGACAAAGGCCGCTTGTAGCCTTCCATCCGTGACCTCAATGCGCTGCTTAACCAGCTCCACCAGGTCGGCGGTGGCCCATATGTCGTAAGGGCTTGGCTGCACCGGAATCAGCACGAAGTCGGCTGCCTTGATCGCGGACACAGCCAAGTCCGCCGCCTGGGGCGCTCCGTCGATCACTACGAAGTCGCGCCGGCCGATGGCCTTCACGTCGCGGTCAATCGTCGGGCGGTCGATGCCGACAACGGTTAGCGGTTGATCTTCCCGCACGGCCGCCCAATCGCGGGCACTGCCCTGGGGATCGGAATCGACTAACAGAACATCGGCCCCGGCGAGTTGCAGGGCGCGGGCTAGATGGGTTGCGATGGTCGTCTTGCCTGACCCGCCTTTCTGGTTAAGTACAGCGATAACCTTCATGCGTTCCCCTTGCGTATTTGTTTATTTACTCATCGCATCATATACGCAGCGACCGCATGACGCAAGCTGTTTTACTCAAATACACATCACCTTTTTAGACGGCGGCGCTCGGTTTCTTCAGCGGCCAAGCTGGCCGGCCAGGCCGCCAGCTTGGCATCAGACAAACCGGCCAGGATTTCATGCAGCCGCACGGTTGAGACGTGCGCGGGCGGCTCGAACACGTACCCGGCCGCGATCATCTCCGCCTCGATCTCTTCGGTAATGAAAAACGGTTCGTCCTGGCCGTCCTGGTGCGGTTTCATGCTTGTTCCTCTTGGCGTTCATTCTCGGCGGCCGCCAGGGCGTCGGCCTCGGTCAATGCGTCCTCACGGAAGGCACCGCGCCGCCTGGCCTCGGTGGGCGTCACTTCCTCGCTGCGCTCAAGTGCGCGGTACAGGGTCGAGCGATGCACGCCAAGCAGTGCAGCCGCCTCTTTCACGGTGCGGCCTTCCTGGTCGATCAGCTCGCGGGCGTGCGCGATCTGTGCCGGGGTGAGGGTAGGGCGGGGGCCAAACTTCACGCCTCGGGCCTTGGCGGCCTCGCGCCCGCTCCGGGTGCGGTCGATGATTAGGGAACGCTCGAACTCGGCAATGCCGGCGAACACGGTCAACACCATGCGGCCGGCCGGCGTGGTGGTGTCGGCCCACGGCTCTGCCAGGCTACGCAGGCCCGCGCCGGCCTCCTGGATGCGCTCGGCAATGTCCAGTAGGTCGCGGGTGCTGCGGGCCAGGCGGTCTAGCCTGGTCACTGTCACAACGTCGCCAGGGCGTAGGTGGTCAAGCATCCTGGCCAGCTCCGGGCGGTCGCGCCTGGTGCCGGTGATCTTCTCGGAAAACAGCTTGGTGCAGCCGGCCGCGTGCAGTTCGGCCCGTTGGTTGGTCAAGTCCTGGTCGTCGGTGCTGACGCGGGCATAGCCCAGCAGGCCAGCGGCGGCGCTCTTGTTCATGGCGTAATGTCTCCGGTTCTAGTCGCAAGTATTCTACTTTATGCGACTAAAACACGCGACAAGAAAACGCCAGGAAAAGGGCAGGGCGGCAGCCTGTCGCGTAACTTAGGACTTGTGCGACATGTCGTTTTCAGAAGACGGCTGCACTGAACGTCAGAAGCCGACTGCACTATAGCAGCGGAGGGGTTGGATCAAAGTACTTTGATCCCGAGGGGAACCCTGTGGTTGGCATGCACATACAAATGGACGAACGGATAAACCTTTTCACGCCCTTTTAAATATCCGTTATTCTAATAAACGCTCTTTTCTCTTAGGTTTACCCGCCAATATATCCTGTCAAACACTGATAGTTTAAACTGAAGGCGGGAAACGACAATCTGATCCAAGCTCAAGCTAAGCTTCAGGAAACAGCTATGACCATGTAATACGACTCACTATAGGGGATATCGCGGCCGCGTTAACGCTACCATGGAGCTCCAAATAATGATTTTATTTTGACTGATAGTGACCTGTTCGTTGCAACAAATTGATAAGCAATGCTTTTTTATAATGCCAACTTTGTATAATAAAGTTGAACGAGAAACGTAAAATGATATAAATATCAATATATTAAATTAGATTTTGCATAAAAAACAGACTACATAATACTGTAAAACACAACATATGCAGTCACTATGAATCAACTACTTAGATGGTATTAGTGACCTGTAGAATTCGAGCTCGACCGACAGCCTTCCAAATGTTCTTCTCAAACGGAATCGTCGTATCCAGCCTACTCGCTATTGTCCTCAATGCCGTATTAAATCATAAAAAGAAATAAGAAAAAGAGGTGCGAGCCTCTTTTTTGTGTGACAAAATAAAAACATCTACCTATTCATATACGCTAGTGTCATAGTCCTGAAAATCATCTGCATCAAGAACAATTTCACAACTCTTATACTTTTCTCTTACAAGTCGTTCGGCTTCATCTGGATTTTCAGCCTCTATACTTACTAAACGTGATAAAGTTTCTGTAATTTCTACTGTATCGACCTGCAGACTGGCTGTGTATAAGGGAGCCTGACATTTATATTCCCCAGAACATCAGGTTAATGGCGTTTTTGATGTCATTTTCGCGGTGGCTGAGATCAGCCACTTCTTCCCCGATAACGGAGACCGGCACACTGGCCATATCGGTGGTCATCATGCGCCAGCTTTCATCCCCGATATGCACCACCGGGTAAAGTTCACGGGAGACTTTATCTGACAGCAGACGTGCACTGGCCAGGGGGATCACCATCCGTCGCCCGGGCGTGTCAATAATATCACTCTGTACATCCACAAACAGACGATAACGGCTCTCTCTTTTATAGGTGTAAACCTTAAACTGCATTTCACCAGTCCCTGTTCTCGTCAGCAAAAGAGCCGTTCATTTCAATAAACCGGGCGACCTCAGCCATCCCTTCCTGATTTTCCGCTTTCCAGCGTTCGGCACGCAGACGACGGGCTTCATTCTGCATGGTTGTGCTTACCAGACCGGAGATATTGACATCATATATGCCTTGAGCAACTGATAGCTGTCGCTGTCAACTGTCACTGTAATACGCTGCTTCATAGCACACCTCTTTTTGACATACTTCGGGTATACATATCAGTATATATTCTTATACCGCAAAAATCAGCGCGCAAATACGCATACTGTTATCTGGCTTTTAGTAAGCCGGATCCACGCGATTACGCCCCGCCCTGCCACTCATCGCAGTACTGTTGTAATTCATTAAGCATTCTGCCGACATGGAAGCCATCACAGACGGCATGATGAACCTGAATCGCCAGCGGCATCAGCACCTTGTCGCCTTGCGTATAATATTTGCCCATGGTGAAAACGGGGGCGAAGAAGTTGTCCATATTGGCCACGTTTAAATCAAAACTGGTGAAACTCACCCAGGGATTGGCTGAGACGAAAAACATATTCTCAATAAACCCTTTAGGGAAATAGGCCAGGTTTTCACCGTAACACGCCACATCTTGCGAATATATGTGTAGAAACTGCCGGAAATCGTCGTGGTATTCACTCCAGAGCGATGAAAACGTTTCAGTTTGCTCATGGAAAACGGTGTAACAAGGGTGAACACTATCCCATATCACCAGCTCACCGTCTTTCATTGCCATACGGAATTCCGGATGAGCATTCATCAGGCGGGCAAGAATGTGAATAAAGGCCGGATAAAACTTGTGCTTATTTTTCTTTACGGTCTTTAAAAAGGCCGTAATATCCAGCTGAACGGTCTGGTTATAGGTACATTGAGCAACTGACTGAAATGCCTCAAAATGTTCTTTACGATGCCATTGGGATATATCAACGGTGGTATATCCAGTGATTTTTTTCTCCATTTTAGCTTCCTTAGCTCCTGAAAATCTCGATAACTCAAAAAATACGCCCGGTAGTGATCTTATTTCATTATGGTGAAAGTTGGAACCTCTTACGTGCCGATCAACGTCTCATTTTCGCCAAAAGTTGGCCCAGGGCTTCCCGGTATCAACAGGGACACCAGGATTTATTTATTCTGCGAAGTGATCTTCCGTCACAGGTATTTATTCGGCGCAAAGTGCGTCGGGTGATGCTGCCAACTTAGTCGACGCTCTAGAGCTCCAAATAATGATTTTATTTTGACTGATAGTGACCTGTTCGTTGCAACAAATTGATAAGCAATGCTTTTTTATAATGCCAACTTTGTACAAGAAAGTTGAACGAGAAACGTAAAATGATATAAATATCAATATATTAAATTAGATTTTGCATAAAAAACAGACTACATAATACTGTAAAACACAACATATGCAGTCACTATGAATCAACTACTTAGATGGTATTAGTGACCTGTAGAATTCGAGCTCTAGAGCTGCAGGGCCCGAGCTTAAGACTGGCCGTCGTTTTACGGTACCGCGATCGCTCGCGACCTGCAGGCATAAAGCCGTCAGTGTCCGCATAAAGAACCACCCATAATACCCATAATAGCTGTTTGCCATCGCTACCTTAGTTATACCTCGTATTCGATTAAATGATACAACTCATTGCCACACCGCAAGACGGCGTCACCACCATAGACAGCCTTTCAAGCACCCCCTTGTCTATGAGCTGCCACGTCAGCTGCCGCTCCTAATATCCACCCGCGTCCCTATTCCCGTCATATGGACGTACTTGATGGTAAAAATCAAGGGCCAACTGACTACCCACGGAAAAATCGCTATCCCATCAAAGCAGTCGAGTTTTTCCTTTTTTTTTCTTATCTTTTTCAAGTCAAATCAAAATGTCGCTACCATAACCATTATACCGCCCTCAAAATAAAGTCTATCCCTCCTATCCACTGGTCGGTTCCTCTCCCTCTGATGGACAAAGGGTCGCCTCTACGTACCCGCGACAATCTCGGCTTTTCCAATGTCGACAGCTGGCATATCGCACGCGAGGTTAGGCGTCTTCAGCCGGCGTAACCACAAACCTCGAGTCAAGTACCCTTTTTCAGACCTTAATTTCCGTGTCCATTCCCTCGCGTTTACACGAAGGTTTCTGAGAAATACCACATGTCACAGAGAGAGAGAAGATTGGAGAACCTTCTCAACCTGACCCGTGCAGAGCGCTTTTGCGTCCTACCATCCGTTAATTATAGCACCACCACCACATGTCTCGCCGGAACTCTCTTATCCGCTCTTTTTGATCGTCTTTTTTGCGCCCCATTAAAGACATCTCTTGCACAGGGATCGTTTCTGATTGGCCATTTCACTTCCTCTTTTACATCCGAAAACCACCTGTTTGTGGGGTTGTGAAGCCACAACCGGACGCTCAAAAAGCCTCGCCCGACTCGCCCACGATCGGAAGGCTCATTCTCCTTTTCACTCTCACGTGCCCAAGTCCCAACCGACTCTTTTTCGACCTTCACTCTCACCGACAACCCGGCTCTAACTATAACGGTCCTAAATGGTGAGCAAGGGCGAGGAGCTGTTCACCGGGGTGGTGCCCATCCTGGTCGAGCTGGACGGCGACGTAAACGGCCACAAGTTCAGCGTGTCCGGCGAGGGCGAGGGCGATGCCACCTACGGCAAGCTGACCCTGAAGTTCATCTGCACCACCGGCAAGCTGCCCGTGCCCTGGCCCACCCTCGTGACCACCCTGACCTACGGCGTGCAGTGCTTCAGCCGCTACCCCGACCACATGAAGCAGCACGACTTCTTCAAGTCCGCCATGCCCGAAGGCTACGTCCAGGAGCGCACCATCTTCTTCAAGGACGACGGCAACTACAAGACCCGCGCCGAGGTGAAGTTCGAGGGCGACACCCTGGTGAACCGCATCGAGCTGAAGGGCATCGACTTCAAGGAGGACGGCAACATCCTGGGGCACAAGCTGGAGTACAACTACAACAGCCACAACGTCTATATCATGGCCGACAAGCAGAAGAACGGCATCAAGGTGAACTTCAAGATCCGCCACAACATCGAGGACGGCAGCGTGCAGCTCGCCGACCACTACCAGCAGAACACCCCCATCGGCGACGGCCCCGTGCTGCTGCCCGACAACCACTACCTGAGCACCCAGTCCGCCCTGAGCAAAGACCCCAACGAGAAGCGCGATCACATGGTCCTGCTGGAGTTCGTGACCGCCGCCGGGATCACTCTCGGCATGGACGAGCTGTACAAGTGACTAAGGTAGCGACCTTGACGTCTAACCGCGGGGTGCCTACTTCTTGTAAGCCGAAGGTCGAGCTGTGTGCACTGAGTGCGAAGTTGCAGATTTTGTGTCTATGCATTTAATACAATTTCCTTTTTTCCATTTTCGTTGTTTTTTTTTGTGAAAGAACGATTGTTTTCAAAAGAAGGCCGTTTGTAGAACAATTTTTTTTTATACTAGCATTTGGTATTCGAGTGTGACGAATCGGTGATGGCGCTTTACAAATGAAATATAACGCTGAACAAGCGCAGTGACAGCTTTATTTTATAACAGAGAATAAAGACCAAAGTAGAGTAGACTGATGGCGGCTTGAAACAAAACTACGTGACTTCTAATGCAAAGAAAACCATACCCCACGCTACTTAGAATTGTTTCTCTTTGGTTGATGACCGCTATAGTCGTATGGAGTGATCCCTTTTTGTCAATATTTCGATTCTTGCTAAAACCGTGACTATTTTGAGGCAACTGTGTGTACTTAACATGGTAGAAATTTCTATGTTTAAGCCAGCTGACAATCATTCTTTATTTATCGTAAACCAACCATTAGGACCGTTATAGTTAATTACCCTGTTATCCCTATTAATTAAGAGCTCGCTACCTTAAGAGAGGATATCGGCGCGTCCGTCATTTCCTCGCAGCAACTCGCTTCCACGATTTCTTTGTATTCACTCGGCAGCCTCGAGCTGATTGAGCGAGAGATGGACGTCCACGCCGATGGATCACCCGTTTGGCCGCAATATTTGCCTGGACTGTTAGATCCCGTACCAGAGATAGACATGGTCAGCGACGGCTTCATTACCTCGTCGGAATCATTGCTGGAACATCAAACAAGTTTTATCTACGGCTTCGATAACCCTGAGTCGACGGCATCATCTGGAGCATTGGTAGATGCTGATGACGTGTGCTCTTCCACCACTTCTAGACTGTGTATCAAGCTCCGGTTCACAGGCGCCGATCAGTTGGCTCCAATATTTCTTCGTAAACTGCAGAGCATTCACGCTGCACTATCTTCGTCAATTGGTCTTCCTCCCGGATCAAGTGTTGCTGGTTCAACTTCTACTTATCCGGCTCCGTGGGTGCTGGTTGGTGCTCAGAGTCTCGGTGGGGATACTTCGTGGTCCTTCTTCGTAGGCGGGTCGTCATCAAGCGGCAATGCCCAGAGCTTCCGTCTCACGTTCGAAGCTGTAAACTCGATGCAAGATATGACCACTACCACACCCTTCACCATAGACCTGGAGTTTGTTCCTGACGACCCAACTGTCATCCCCATTGTGCGACGTCGGCAGTTTGGCGACCTCGCGGTGGTAGACACCACTGGCCAGCCTGAACCAGCATTTATCACCTCTGAGATTCCTGATCTGATCGACGATGGAGACGAAGTCACTGGTATCACCATAACATTGGATTTGGAGCAGCCTCGACTTGGTGGACTGTATTCAGGTTCACAACTGGATGATGTAGCCCCAACTTGCAGCGAGTGCACGACGTTATTGGAGGATTGCAGCAGCTCGCCCGAGTGCCGAGCGTTCTCAGCGTGTATGGGTGTTCTGCTTGAGGCAGACTTGACACTGATAGCTACGATGCTGGAGAAGGACGATATCGACAGCAGCGTTGATGCAACTTGGCTGCTGCAGGACTGCTTGAGTCCATCGGACGGCACGGAATGGTCGAGTACAGTGCGTGAGCTGCTCGAGTCCAGCTACGCTTGTCTGTGGGCGAAGAGCTGTCCACTGGCATACAGCACCACAATAGGGAGGCAGATCGTGCTGGACTACAGTCCTGGAGAGCAGATCTTGACCTTCGACCTGGCGAGCGGATTCGCCAGCGTCAGCTTCGAGCTCGGCAACTGGGCGTACAGTTTTGTCGAGGACTTCACGGCGAACGCGACGGAAGTGACAGCCCAACTGGACACGATGCTGATGGAGATGTACCGCACTGCGCTGTCGAATGTGGCGACGGTGAAGTCGGCCCTCGTCACGACGGAAGACTCGAACACGGGAGTGGTGACGGCGCAGTTGACCATTCTCTACGAGTTCCTGGGCGCACTGCCACAGCCTTATGTCGCAGAGAATGGAGGTTCGTTGGCGTGCAATACCACCGAGGAGGCTCTGCATCTGCGCGTACTAGCCATAACGTAAGAACGAGTGCAATAACAAGAACAACTTCACTTGCAGTGCAAGTTGAAGACACTATAGTGGGGAAAGTGTCACTCAAGTGGGGTCACATTTTGGTTGGCCAGTAGTCCGCGCCCGCTGTCCAGCCAATCAAAGTGTTTGAAGATTCCATCTCCTCTTTTTTGCACCATACAACGCCCTTCATGCGGGACTTGTTGCCTACAATAACCAAGTCTCTCCACGCTGGCAAGCTCGCAGCCAACTATCCTCTCGCATGTTGATCCCAAGGCTGCGCTCGCCGACAGCAACAAGCCTCACTTTCTGCTGACTGCTCTGCTTGGAACATCGTTCGGGGTAAGTGGCGACCCCCTTGTCTCTCTCTGAGCCTCGCAGCTATTGGCCCCACATGGCTACATCGACAGTCTTTACTTACTATAGTTTTAATTCGTTCGGTGACGCTAGCATCTGAGCATCCACCCGGGCAACAATGATTGAACAAGATGGATTGCACGCAGGTTCTCCGGCCGCTTGGGTGGAGAGGCTATTCGGCTATGACTGGGCACAACAGACAATCGGCTGCTCTGATGCCGCCGTGTTCCGGCTGTCAGCGCAGGGGCGCCCGGTTCTTTTTGTCAAGACCGACCTGTCCGGTGCCCTGAATGAACTGCAGGACGAGGCAGCGCGGCTATCGTGGCTGGCCACGACGGGCGTTCCTTGCGCAGCTGTGCTCGACGTTGTCACTGAAGCGGGAAGGGACTGGCTGCTATTGGGCGAAGTGCCGGGGCAGGATCTCCTGTCATCTCACCTTGCTCCTGCCGAGAAAGTATCCATCATGGCTGATGCAATGCGGCGGCTGCATACGCTTGATCCGGCTACCTGCCCATTCGACCACCAAGCGAAACATCGCATCGAGCGAGCACGTACTCGGATGGAAGCCGGTCTTGTCGATCAGGATGATCTGGACGAAGAGCATCAGGGGCTCGCGCCAGCCGAACTGTTCGCCAGGCTCAAGGCGCGCATGCCCGACGGCGAGGATCTCGTCGTGACCCATGGCGATGCCTGCTTGCCGAATATCATGGTGGAAAATGGCCGCTTTTCTGGATTCATCGACTGTGGCCGGCTGGGTGTGGCGGACCGCTATCAGGACATAGCGTTGGCTACCCGTGATATTGCTGAAGAGCTTGGCGGCGAATGGGCTGACCGCTTCCTCGTGCTTTACGGTATCGCCGCTCCCGATTCGCAGCGCATCGCCTTCTATCGCCTTCTTGACGAGTTCTTCTGATAGGTACCGGTGTCGACTAGAATACTTAGCAATTGGTTAGTTTGAAAATTGTCAAAGCAGAGTTTTTTCGACAATAAAATTTATTTTCTAAATATTTGATTTAGCGTTGACTTAATTTGTTGATTCGGTTGTCATCTTTTGTGAAGATGCCCGAGAGTCTAGTAAATCTCAAGCGAAACATAGTGAGAAGGTCACTATGGCTGGTTTGCATGAGTCTGGGCCTATTGTGCTTACGCCGCCTTAGTAACAAACATGCCAAAAGAAGGAAAGCTTTCACCAGCTTTCAAGTTTAATCAATCTTTGAGAGTCACAATTGCAAGAATAAATGAATTTTTACAACACAGTTCGATGCACTGTGTTTCGTCTTGATGAAAATTGGACGGCAAATCGAGTATCTTAAACAAATCTCGGCATAGGCTGTCCATTTTGTGGTCGCCTCTTTGTTGGTAGTTTGCAGTAGTTTCATTAGTAGAAGATCTGCATCCTCAATCTGCTAAACGTCTTGAGCAGCTATGAGACGATCCGGGGAATTGGGGCGCGCCTCTAGAATTTAAATGGATCCTACGTACTCGAGGAATTCAATTCGGCGTTAATTCAGTACATTAAAAACGTCCGCAATGTGTTATTAAGTTGTCTAAGCGTCAATTTGTTTACACCACAATATATCCTGCCACCAGCCAGCCAACAGCTCCCCGACCGGCAGCTCGGCACAAAATCACCACTCGATACAGGCAGCCCATCAGTCCGGGACGGCGTCAGCGGGAGAGCCGTTGTAAGGCGGCAGACTTTGCTCATGTTACCGATGCTATTCGGAAGAACGGCAACTAAGCTGCCGGGTTTGAAACACGGATGATCTCGCGGAGGGTAGCATGTTGATTGTAACGATGACAGAGCGTTGCTGCCTGTGATCAATTCGGGCACGAACCCAGTGGACATAAGCCTCGTTCGGTTCGTAAGCTGTAATGCAAGTAGCGTAACTGCCGTCACGCAACTGGTCCAGAACCTTGACCGAACGCAGCGGTGGTAACGGCGCAGTGGCGGTTTTCATGGCTTCTTGTTATGACATGTTTTTTTGGGGTACAGTCTATGCCTCGGGCATCCAAGCAGCAAGCGCGTTACGCCGTGGGTCGATGTTTGATGTTATGGAGCAGCAACGATGTTACGCAGCAGGGCAGTCGCCCTAAAACAAAGTTAAACATCATGGGGGAAGCGGTGATCGCCGAAGTATCGACTCAACTATCAGAGGTAGTTGGCGTCATCGAGCGCCATCTCGAACCGACGTTGCTGGCCGTACATTTGTACGGCTCCGCAGTGGATGGCGGCCTGAAGCCACACAGTGATATTGATTTGCTGGTTACGGTGACCGTAAGGCTTGATGAAACAACGCGGCGAGCTTTGATCAACGACCTTTTGGAAACTTCGGCTTCCCCTGGAGAGAGCGAGATTCTCCGCGCTGTAGAAGTCACCATTGTTGTGCACGACGACATCATTCCGTGGCGTTATCCAGCTAAGCGCGAACTGCAATTTGGAGAATGGCAGCGCAATGACATTCTTGCAGGTATCTTCGAGCCAGCCACGATCGACATTGATCTGGCTATCTTGCTGACAAAAGCAAGAGAACATAGCGTTGCCTTGGTAGGTCCAGCGGCGGAGGAACTCTTTGATCCGGTTCCTGAACAGGATCTATTTGAGGCGCTAAATGAAACCTTAACGCTATGGAACTCGCCGCCCGACTGGGCTGGCGATGAGCGAAATGTAGTGCTTACGTTGTCCCGCATTTGGTACAGCGCAGTAACCGGCAAAATCGCGCCGAAGGATGTCGCTGCCGACTGGGCAATGGAGCGCCTGCCGGCCCAGTATCAGCCCGTCATACTTGAAGCTAGACAGGCTTATCTTGGACAAGAAGAAGATCGCTTGGCCTCGCGCGCAGATCAGTTGGAAGAATTTGTCCACTACGTGAAAGGCGAGATCACCAAGGTAGTCGGCAAATAATGTCTAGCTAGAAATTCGTTCAAGCCGACGCCGCTTCGCCGGCGTTAACTCAAGCGATTAGATGCACTAAGCACATAATTGCTCACAGCCAAACTATCAGGTCAAGTCTGCTTTTATTATTTTTAAGCGTGCATAATAAGCCCTACACAAATTGGGAGATATATCATGCATGACCAAAATCCCTTAACGTGAGTTTTCGTTCCACTGAGCGTCAGACCCCGTAGAAAAGATCAAAGGATCTTCTTGAGATCCTTTTTTTCTGCGCGTAATCTGCTGCTTGCAAACAAAAAAACCACCGCTACCAGCGGTGGTTTGTTTGCCGGATCAAGAGCTACCAACTCTTTTTCCGAAGGTAACTGGCTTCAGCAGAGCGCAGATACCAAATACTGTCCTTCTAGTGTAGCCGTAGTTAGGCCACCACTTCAAGAACTCTGTAGCACCGCCTACATACCTCGCTCTGCTAATCCTGTTACCAGTGGCTGCTGCCAGTGGCGATAAGTCGTGTCTTACCGGGTTGGACTCAAGACGATAGTTACCGGATAAGGCGCAGCGGTCGGGCTGAACGGGGGGTTCGTGCACACAGCCCAGCTTGGAGCGAACGACCTACACCGAACTGAGATACCTACAGCGTGAGCTATGAGAAAGCGCCACGCTTCCCGAAGGGAGAAAGGCGGACAGGTATCCGGTAAGCGGCAGGGTCGGAACAGGAGAGCGCACGAGGGAGCTTCCAGGGGGAAACGCCTGGTATCTTTATAGTCCTGTCGGGTTTCGCCACCTCTGACTTGAGCGTCGATTTTTGTGATGCTCGTCAGGGGGGCGGAGCCTATGGAAAAACGCCAGCAACGCGGCCTTTTTACGGTTCCTGGCCTTTTGCTGGCCTTTTGCTCACATGTTCTTTCCTGCGTTATCCCCTGATTCTGTGGATAACCGTATTACCGCCTTTGAGTGAGCTGATACCGCTCGCCGCAGCCGAACGACCGAGCGCAGCGAGTCAGTGAGCGAGGAAGCGGAAGAGCGCCTGATGCGGTATTTTCTCCTTACGCATCTGTGCGGTATTTCACACCGCATATGGTGCACTCTCAGTACAATCTGCTCTGATGCCGCATAGTTAAGCCAGTATACACTCCGCTATCGCTACGTGACTGGGTCATGGCTGCGCCCCGACACCCGCCAACACCCGCTGACGCGCCCTGACGGGCTTGTCTGCTCCCGGCATCCGCTTACAGACAAGCTGTGACCGTCTCCGGGAGCTGCATGTGTCAGAGGTTTTCACCGTCATCACCGAAACGCGCGAGGCAGGGTGCCTTGATGTGGGCGCCGGCGGTCGAGTGGCGACGGCGCGGCTTGTCCGCGCCCTGGTAGATTGCCTGGCCGTAGGCCAGCCATTTTTGAGCGGCCAGCGGCCGCGATAGGCCGACGCGAAGCGGCGGGGCGTAGGGAGCGCAGCGACCGAAGGGTAGGCGCTTTTTGCAGCTCTTCGGCTGTGCGCTGGCCAGACAGTTATGCACAGGCCAGGCGGGTTTTAAGAGTTTTAATAAGTTTTAAAGAGTTTTAGGCGGAAAAATCGCCTTTTTTCTCTTTTATATCAGTCACTTACATGTGTGACCGGTTCCCAATGTACGGCTTTGGGTTCCCAATGTACGGGTTCCGGTTCCCAATGTACGGCTTTGGGTTCCCAATGTACGTGCTATCCACAGGAAAGAGACCTTTTCGACCTTTTTCCCCTGCTAGGGCAATTTGCCCTAGCATCTGCTCCGTACATTAGGAACCGGCGGATGCTTCGCCCTCGATCAGGTTGCGGTAGCGCATGACTAGGATCGGGCCAGCCTGCCCCGCCTCCTCCTTCAAATCGTACTCCGGCAGGTCATTTGACCCGATCAGCTTGCGCACGGTGAAACAGAACTTCTTGAACTCTCCGGCGCTGCCACTGCGTTCGTAGATCGTCTTGAACAACCATCTGGCTTCTGCCTTGCCTGCGGCGCGGCGTGCCAGGCGGTAGAGAAAACGGCCGATGCCGGGATCGATCAAAAAGTAATCGGGGTGAACCGTCAGCACGTCCGGGTTCTTGCCTTCTGTGATCTCGCGGTACATCCAATCAGCTAGCTCGATCTCGATGTACTCCGGCCGCCCGGTTTCGCTCTTTACGATCTTGTAGCGGCTAATCAAGGCTTCACCCTCGGATACCGTCACCAGGCGGCCGTTCTTGGCCTTCTTCGTACGCTGCATGGCAACGTGCGTGGTGTTTAACCGAATGCAGGTTTCTACCAGGTCGTCTTTCTGCTTTCCGCCATCGGCTCGCCGGCAGAACTTGAGTACGTCCGCAACGTGTGGACGGAACACGCGGCCGGGCTTGTCTCCCTTCCCTTCCCGGTATCGGTTCATGGATTCGGTTAGATGGGAAACCGCCATCAGTACCAGGTCGTAATCCCACACACTGGCCATGCCGGCCGGCCCTGCGGAAACCTCTACGTGCCCGTCTGGAAGCTCGTAGCGGATCACCTCGCCAGCTCGTCGGTCACGCTTCGACAGACGGAAAACGGCCACGTCCATGATGCTGCGACTATCGCGGGTGCCCACGTCATAGAGCATCGGAACGAAAAAATCTGGTTGCTCGTCGCCCTTGGGCGGCTTCCTAATCGACGGCGCACCGGCTGCCGGCGGTTGCCGGGATTCTTTGCGGATTCGATCAGCGGCCGCTTGCCACGATTCACCGGGGCGTGCTTCTGCCTCGATGCGTTGCCGCTGGGCGGCCTGCGCGGCCTTCAACTTCTCCACCAGGTCATCACCCAGCGCCGCGCCGATTTGTACCGGGCCGGATGGTTTGCGACCGTCACGCCGATTCCTCGGGCTTGGGGGTTCCAGTGCCATTGCAGGGCCGGCAGACAACCCAGCCGCTTACGCCTGGCCAACCGCCCGTTCCTCCACACATGGGGCATTCCACGGCGTCGGTGCCTGGTTGTTCTTGATTTTCCATGCCGCCTCCTTTAGCCGCTAAAATTCATCTACTCATTTATTCATTTGCTCATTTACTCTGGTAGCTGCGCGATGTATTCAGATAGCAGCTCGGTAATGGTCTTGCCTTGGCGTACCGCGTACATCTTCAGCTTGGTGTGATCCTCCGCCGGCAACTGAAAGTTGACCCGCTTCATGGCTGGCGTGTCTGCCAGGCTGGCCAACGTTGCAGCCTTGCTGCTGCGTGCGCTCGGACGGCCGGCACTTAGCGTGTTTGTGCTTTTGCTCATTTTCTCTTTACCTCATTAACTCAAATGAGTTTTGATTTAATTTCAGCGGCCAGCGCCTGGACCTCGCGGGCAGCGTCGCCCTCGGGTTCTGATTCAAGAACGGTTGTGCCGGCGGCGGCAGTGCCTGGGTAGCTCACGCGCTGCGTGATACGGGACTCAAGAATGGGCAGCTCGTACCCGGCCAGCGCCTCGGCAACCTCACCGCCGT

> pLY40-Cas12a-gfp

CGGATAAACCTTTTCACGCCCTTTTAAATATCCGATTATTCTAATAAACGCTCTTTTCTCTTAGgTTTACCCGCCAATATATCCTGTCAAACACTGATAGTTTAAACTGAAGGCGGGAAACGACAATCTGATCCAAGCTCAAGCTAAGCTTAGGCCTGTCGACGCCCGGGCGGTACCGCGATCGCTCGCGACCTGCAGGCATAAAGCCGTCAGTGTCCGCATAAAGAACCACCCATAATACCCATAATAGCTGTTTGCCATCGCTACCTTAGTTATACCTCGTATTCGATTAAATGATACAACTCATTGCCACACCGCAAGACGGCGTCACCACCATAGACAGCCTTTCAAGCACCCCCTTGTCTATGAGCTGCCACGTCAGCTGCCGCTCCTAATATCCACCCGCGTCCCTATTCCCGTCATATGGACGTACTTGATGGTAAAAATCAAGGGCCAACTGACTACCCACGGAAAAATCGCTATCCCATCAAAGCAGTCGAGTTTTTCCTTTTTTTTTCTTATCTTTTTCAAGTCAAATCAAAATGTCGCTACCATAACCATTATACCGCCCTCAAAATAAAGTCTATCCCTCCTATCCACTGGTCGGTTCCTCTCCCTCTGATGGACAAAGGGTCGCCTCTACGTACCCGCGACAATCTCGGCTTTTCCAATGTCGACAGCTGGCATATCGCACGCGAGGTTAGGCGTCTTCAGCCGGCGTAACCACAAACCTCGAGTCAAGTACCCTTTTTCAGACCTTAATTTCCGTGTCCATTCCCTCGCGTTTACACGAAGGTTTCTGAGAAATACCACATGTCACAGAGAGAGAGAAGATTGGAGAACCTTCTCAACCTGACCCGTGCAGAGCGCTTTTGCGTCCTACCATCCGTTAATTATAGCACCACCACCACATGTCTCGCCGGAACTCTCTTATCCGCTCTTTTTGATCGTCTTTTTTGCGCCCCATTAAAGACATCTCTTGCACAGGGATCGTTTCTGATTGGCCATTTCACTTCCTCTTTTACATCCGAAAACCACCTGTTTGTGGGGTTGTGAAGCCACAACCGGACGCTCAAAAAGCCTCGCCCGACTCGCCCACGATCGGAAGGCTCATTCTCCTTTTCACTCTCACGTGCCCAAGTCCCAACCGACTCTTTTTCGACCTTCACTCTCACCGACAACCCGGCTCTAACTATAACGGTCCTAAATGAGCAAGCTGGAGAAGTTTACAAACTGCTACTCCCTGTCTAAGACCCTGAGGTTCAAGGCCATCCCTGTGGGCAAGACCCAGGAGAACATCGACAATAAGCGGCTGCTGGTGGAGGACGAGAAGAGAGCCGAGGATTATAAGGGCGTGAAGAAGCTGCTGGATCGCTACTATCTGTCTTTTATCAACGACGTGCTGCACAGCATCAAGCTGAAGAATCTGAACAATTACATCAGCCTGTTCCGGAAGAAAACCAGAACCGAGAAGGAGAATAAGGAGCTGGAGAACCTGGAGATCAATCTGCGGAAGGAGATCGCCAAGGCCTTCAAGGGCAACGAGGGCTACAAGTCCCTGTTTAAGAAGGATATCATCGAGACAATCCTGCCAGAGTTCCTGGACGATAAGGACGAGATCGCCCTGGTGAACAGCTTCAATGGCTTTACCACAGCCTTCACCGGCTTCTTTGATAACAGAGAGAATATGTTTTCCGAGGAGGCCAAGAGCACATCCATCGCCTTCAGGTGTATCAACGAGAATCTGACCCGCTACATCTCTAATATGGACATCTTCGAGAAGGTGGACGCCATCTTTGATAAGCACGAGGTGCAGGAGATCAAGGAGAAGATCCTGAACAGCGACTATGATGTGGAGGATTTCTTTGAGGGCGAGTTCTTTAACTTTGTGCTGACACAGGAGGGCATCGACGTGTATAACGCCATCATCGGCGGCTTCGTGACCGAGAGCGGCGAGAAGATCAAGGGCCTGAACGAGTACATCAACCTGTATAATCAGAAAACCAAGCAGAAGCTGCCTAAGTTTAAGCCACTGTATAAGCAGGTGCTGAGCGATCGGGAGTCTCTGAGCTTCTACGGCGAGGGCTATACATCCGATGAGGAGGTGCTGGAGGTGTTTAGAAACACCCTGAACAAGAACAGCGAGATCTTCAGCTCCATCAAGAAGCTGGAGAAGCTGTTCAAGAATTTTGACGAGTACTCTAGCGCCGGCATCTTTGTGAAGAACGGCCCCGCCATCAGCACAATCTCCAAGGATATCTTCGGCGAGTGGAACGTGATCCGGGACAAGTGGAATGCCGAGTATGACGATATCCACCTGAAGAAGAAGGCCGTGGTGACCGAGAAGTACGAGGACGATCGGAGAAAGTCCTTCAAGAAGATCGGCTCCTTTTCTCTGGAGCAGCTGCAGGAGTACGCCGACGCCGATCTGTCTGTGGTGGAGAAGCTGAAGGAGATCATCATCCAGAAGGTGGATGAGATCTACAAGGTGTATGGCTCCTCTGAGAAGCTGTTCGACGCCGATTTTGTGCTGGAGAAGAGCCTGAAGAAGAACGACGCCGTGGTGGCCATCATGAAGGACCTGCTGGATTCTGTGAAGAGCTTCGAGAATTACATCAAGGCCTTCTTTGGCGAGGGCAAGGAGACAAACAGGGACGAGTCCTTCTATGGCGATTTTGTGCTGGCCTACGACATCCTGCTGAAGGTGGACCACATCTACGATGCCATCCGCAATTATGTGACCCAGAAGCCCTACTCTAAGGATAAGTTCAAGCTGTATTTTCAGAACCCTCAGTTCATGGGCGGCTGGGACAAGGATAAGGAGACAGACTATCGGGCCACCATCCTGAGATACGGCTCCAAGTACTATCTGGCCATCATGGATAAGAAGTACGCCAAGTGCCTGCAGAAGATCGACAAGGACGATGTGAACGGCAATTACGAGAAGATCAACTATAAGCTGCTGCCCGGCCCTAATAAGATGCTGCCAAAGGTGTTCTTTTCTAAGAAGTGGATGGCCTACTATAACCCCAGCGAGGACATCCAGAAGATCTACAAGAATGGCACATTCAAGAAGGGCGATATGTTTAACCTGAATGACTGTCACAAGCTGATCGACTTCTTTAAGGATAGCATCTCCCGGTATCCAAAGTGGTCCAATGCCTACGATTTCAACTTTTCTGAGACAGAGAAGTATAAGGACATCGCCGGCTTTTACAGAGAGGTGGAGGAGCAGGGCTATAAGGTGAGCTTCGAGTCTGCCAGCAAGAAGGAGGTGGATAAGCTGGTGGAGGAGGGCAAGCTGTATATGTTCCAGATCTATAACAAGGACTTTTCCGATAAGTCTCACGGCACACCCAATCTGCACACCATGTACTTCAAGCTGCTGTTTGACGAGAACAATCACGGACAGATCAGGCTGAGCGGAGGAGCAGAGCTGTTCATGAGGCGCGCCTCCCTGAAGAAGGAGGAGCTGGTGGTGCACCCAGCCAACTCCCCTATCGCCAACAAGAATCCAGATAATCCCAAGAAAACCACAACCCTGTCCTACGACGTGTATAAGGATAAGAGGTTTTCTGAGGACCAGTACGAGCTGCACATCCCAATCGCCATCAATAAGTGCCCCAAGAACATCTTCAAGATCAATACAGAGGTGCGCGTGCTGCTGAAGCACGACGATAACCCCTATGTGATCGGCATCGATAGGGGCGAGCGCAATCTGCTGTATATCGTGGTGGTGGACGGCAAGGGCAACATCGTGGAGCAGTATTCCCTGAACGAGATCATCAACAACTTCAACGGCATCAGGATCAAGACAGATTACCACTCTCTGCTGGACAAGAAGGAGAAGGAGAGGTTCGAGGCCCGCCAGAACTGGACCTCCATCGAGAATATCAAGGAGCTGAAGGCCGGCTATATCTCTCAGGTGGTGCACAAGATCTGCGAGCTGGTGGAGAAGTACGATGCCGTGATCGCCCTGGAGGACCTGAACTCTGGCTTTAAGAATAGCCGCGTGAAGGTGGAGAAGCAGGTGTATCAGAAGTTCGAGAAGATGCTGATCGATAAGCTGAACTACATGGTGGACAAGAAGTCTAATCCTTGTGCAACAGGCGGCGCCCTGAAGGGCTATCAGATCACCAATAAGTTCGAGAGCTTTAAGTCCATGTCTACCCAGAACGGCTTCATCTTTTACATCCCTGCCTGGCTGACATCCAAGATCGATCCATCTACCGGCTTTGTGAACCTGCTGAAAACCAAGTATACCAGCATCGCCGATTCCAAGAAGTTCATCAGCTCCTTTGACAGGATCATGTACGTGCCCGAGGAGGATCTGTTCGAGTTTGCCCTGGACTATAAGAACTTCTCTCGCACAGACGCCGATTACATCAAGAAGTGGAAGCTGTACTCCTACGGCAACCGGATCAGAATCTTCCGGAATCCTAAGAAGAACAACGTGTTCGACTGGGAGGAGGTGTGCCTGACCAGCGCCTATAAGGAGCTGTTCAACAAGTACGGCATCAATTATCAGCAGGGCGATATCAGAGCCCTGCTGTGCGAGCAGTCCGACAAGGCCTTCTACTCTAGCTTTATGGCCCTGATGAGCCTGATGCTGCAGATGCGGAACAGCATCACAGGCCGCACCGACGTGGATTTTCTGATCAGCCCTGTGAAGAACTCCGACGGCATCTTCTACGATAGCCGGAACTATGAGGCCCAGGAGAATGCCATCCTGCCAAAGAACGCCGACGCCAATGGCGCCTATAACATCGCCAGAAAGGTGCTGTGGGCCATCGGCCAGTTCAAGAAGGCCGAGGACGAGAAGCTGGATAAGGTGAAGATCGCCATCTCTAACAAGGAGTGGCTGGAGTACGCCCAGACCAGCGTGAAGCACATGGTGAGCAAGGGCGAGGAGCTGTTCACCGGGGTGGTGCCCATCCTGGTCGAGCTGGACGGCGACGTAAACGGCCACAAGTTCAGCGTGTCCGGCGAGGGCGAGGGCGATGCCACCTACGGCAAGCTGACCCTGAAGTTCATCTGCACCACCGGCAAGCTGCCCGTGCCCTGGCCCACCCTCGTGACCACCCTGACCTACGGCGTGCAGTGCTTCAGCCGCTACCCCGACCACATGAAGCAGCACGACTTCTTCAAGTCCGCCATGCCCGAAGGCTACGTCCAGGAGCGCACCATCTTCTTCAAGGACGACGGCAACTACAAGACCCGCGCCGAGGTGAAGTTCGAGGGCGACACCCTGGTGAACCGCATCGAGCTGAAGGGCATCGACTTCAAGGAGGACGGCAACATCCTGGGGCACAAGCTGGAGTACAACTACAACAGCCACAACGTCTATATCATGGCCGACAAGCAGAAGAACGGCATCAAGGTGAACTTCAAGATCCGCCACAACATCGAGGACGGCAGCGTGCAGCTCGCCGACCACTACCAGCAGAACACCCCCATCGGCGACGGCCCCGTGCTGCTGCCCGACAACCACTACCTGAGCACCCAGTCCGCCCTGAGCAAAGACCCCAACGAGAAGCGCGATCACATGGTCCTGCTGGAGTTCGTGACCGCCGCCGGGATCACTCTCGGCATGGACGAGCTGTACAAGTGATCAGCCTAGGCGGACGCGTCTAAGGTAGCGACCTTGACGTCTAACCGCGGGGTGCCTACTTCTTGTAAGCCGAAGGTCGAGCTGTGTGCACTGAGTGCGAAGTTGCAGATTTTGTGTCTATGCATTTAATACAATTTCCTTTTTTCCATTTTCGTTGTTTTTTTTGTGAAAGAACGATTGTTTTCAAAAGAAGGCCGTTTGTAGAACAATTTTTTTTTATACTAGCATTTGGTATTCGAGTGTGACGAATCGGTGATGGCGCTTTACAAATGAAATATAACGCTGAACAAGCGCAGTGACAGCTTTATTTTATAACAGAGAATAAAGACCAAAGTAGAGTAGACTGATGGCGGCTTGAAACAAAACTACGTGACTTCTAATGCAAAGAAAACCATACCCCACGCTACTTAGAATTGTTTCTCTTTGGTTGATGACCGCTATAGTCGTATGGAGTGATCCCTTTTTGTCAATATTTCGATTCTTGCTAAAACCGTGACTATTTTGAGGCAACTGTGTGTACTTAACATGGTAGAAATTTCTATGTTTAAGCCAGCTGACAATCATTCTTTATTTATCGTAAACCAACCATTAGGACCGTTATAGTTAATTACCCTGTTATCCCTATTAATTAAGAGCTCGCTACCTTAAGAGAGGATATCGGCGCGTCCGTCATTTCCTCGCAGCAACTCGCTTCCACGATTTCTTTGTATTCACTCGGCAGCCTCGAGCTGATTGAGCGAGAGATGGACGTCCACGCCGATGGATCACCCGTTTGGCCGCAATATTTGCCTGGACTGTTAGATCCCGTACCAGAGATAGACATGGTCAGCGACGGCTTCATTACCTCGTCGGAATCATTGCTGGAACATCAAACAAGTTTTATCTACGGCTTCGATAACCCTGAGTCGACGGCATCATCTGGAGCATTGGTAGATGCTGATGACGTGTGCTCTTCCACCACTTCTAGACTGTGTATCAAGCTCCGGTTCACAGGCGCCGATCAGTTGGCTCCAATATTTCTTCGTAAACTGCAGAGCATTCACGCTGCACTATCTTCGTCAATTGGTCTTCCTCCCGGATCAAGTGTTGCTGGTTCAACTTCTACTTATCCGGCTCCGTGGGTGCTGGTTGGTGCTCAGAGTCTCGGTGGGGATACTTCGTGGTCCTTCTTCGTAGGCGGGTCGTCATCAAGCGGCAATGCCCAGAGCTTCCGTCTCACGTTCGAAGCTGTAAACTCGATGCAAGATATGACCACTACCACACCCTTCACCATAGACCTGGAGTTTGTTCCTGACGACCCAACTGTCATCCCCATTGTGCGACGTCGGCAGTTTGGCGACCTCGCGGTGGTAGACACCACTGGCCAGCCTGAACCAGCATTTATCACCTCTGAGATTCCTGATCTGATCGACGATGGAGACGAAGTCACTGGTATCACCATAACATTGGATTTGGAGCAGCCTCGACTTGGTGGACTGTATTCAGGTTCACAACTGGATGATGTAGCCCCAACTTGCAGCGAGTGCACGACGTTATTGGAGGATTGCAGCAGCTCGCCCGAGTGCCGAGCGTTCTCAGCGTGTATGGGTGTTCTGCTTGAGGCAGACTTGACACTGATAGCTACGATGCTGGAGAAGGACGATATCGACAGCAGCGTTGATGCAACTTGGCTGCTGCAGGACTGCTTGAGTCCATCGGACGGCACGGAATGGTCGAGTACAGTGCGTGAGCTGCTCGAGTCCAGCTACGCTTGTCTGTGGGCGAAGAGCTGTCCACTGGCATACAGCACCACAATAGGGAGGCAGATCGTGCTGGACTACAGTCCTGGAGAGCAGATCTTGACCTTCGACCTGGCGAGCGGATTCGCCAGCGTCAGCTTCGAGCTCGGCAACTGGGCGTACAGTTTTGTCGAGGACTTCACGGCGAACGCGACGGAAGTGACAGCCCAACTGGACACGATGCTGATGGAGATGTACCGCACTGCGCTGTCGAATGTGGCGACGGTGAAGTCGGCCCTCGTCACGACGGAAGACTCGAACACGGGAGTGGTGACGGCGCAGTTGACCATTCTCTACGAGTTCCTGGGCGCACTGCCACAGCCTTATGTCGCAGAGAATGGAGGTTCGTTGGCGTGCAATACCACCGAGGAGGCTCTGCATCTGCGCGTACTAGCCATAACGTAAGAACGAGTGCAATAACAAGAACAACTTCACTTGCAGTGCAAGTTGAAGACACTATAGTGGGGAAAGTGTCACTCAAGTGGGGTCACATTTTGGTTGGCCAGTAGTCCGCGCCCGCTGTCCAGCCAATCAAAGTGTTTGAAGATTCCATCTCCTCTTTTTTGCACCATACAACGCCCTTCATGCGGGACTTGTTGCCTACAATAACCAAGTCTCTCCACGCTGGCAAGCTCGCAGCCAACTATCCTCTCGCATGTTGATCCCAAGGCTGCGCTCGCCGACAGCAACAAGCCTCACTTTCTGCTGACTGCTCTGCTTGGAACATCGTTCGGGGTAAGTGGCGACCCCCTTGTCTCTCTCTGAGCCTCGCAGCTATTGGCCCCACATGGCTACATCGACAGTCTTTACTTACTATAGTTTTAATTCGTTCGGTGACGCTAGCATCTGAGCATCCACCCGGGCAACAATGATTGAACAAGATGGATTGCACGCAGGTTCTCCGGCCGCTTGGGTGGAGAGGCTATTCGGCTATGACTGGGCACAACAGACAATCGGCTGCTCTGATGCCGCCGTGTTCCGGCTGTCAGCGCAGGGGCGCCCGGTTCTTTTTGTCAAGACCGACCTGTCCGGTGCCCTGAATGAACTGCAGGACGAGGCAGCGCGGCTATCGTGGCTGGCCACGACGGGCGTTCCTTGCGCAGCTGTGCTCGACGTTGTCACTGAAGCGGGAAGGGACTGGCTGCTATTGGGCGAAGTGCCGGGGCAGGATCTCCTGTCATCTCACCTTGCTCCTGCCGAGAAAGTATCCATCATGGCTGATGCAATGCGGCGGCTGCATACGCTTGATCCGGCTACCTGCCCATTCGACCACCAAGCGAAACATCGCATCGAGCGAGCACGTACTCGGATGGAAGCCGGTCTTGTCGATCAGGATGATCTGGACGAAGAGCATCAGGGGCTCGCGCCAGCCGAACTGTTCGCCAGGCTCAAGGCGCGCATGCCCGACGGCGAGGATCTCGTCGTGACCCATGGCGATGCCTGCTTGCCGAATATCATGGTGGAAAATGGCCGCTTTTCTGGATTCATCGACTGTGGCCGGCTGGGTGTGGCGGACCGCTATCAGGACATAGCGTTGGCTACCCGTGATATTGCTGAAGAGCTTGGCGGCGAATGGGCTGACCGCTTCCTCGTGCTTTACGGTATCGCCGCTCCCGATTCGCAGCGCATCGCCTTCTATCGCCTTCTTGACGAGTTCTTCTGATAGGTACCGGTGTCGACTAGAATACTTAGCAATTGGTTAGTTTGAAAATTGTCAAAGCAGAGTTTTTTCGACAATAAAATTTATTTTCTAAATATTTGATTTAGCGTTGACTTAATTTGTTGATTCGGTTGTCATCTTTTGTGAAGATGCCCGAGAGTCTAGTAAATCTCAAGCGAAACATAGTGAGAAGGTCACTATGGCTGGTTTGCATGAGTCTGGGCCTATTGTGCTTACGCCGCCTTAGTAACAAACATGCCAAAAGAAGGAAAGCTTTCACCAGCTTTCAAGTTTAATCAATCTTTGAGAGTCACAATTGCAAGAATAAATGAATTTTTACAACACAGTTCGATGCACTGTGTTTCGTCTTGATGAAAATTGGACGGCAAATCGAGTATCTTAAACAAATCTCGGCATAGGCTGTCCATTTTGTGGTCGCCTCTTTGTTGGTAGTTTGCAGTAGTTTCATTAGTAGAAGATCTGCATCCTCAATCTGCTAAACGTCTTGAGCAGCTATGAGACGATCCGGGGAATTGGGGCGCGCCTCTAGAATTTAAATGGATCCTACGTACTCGAGGAATTCAATTCGGCGTTAATTCAGTACATTAAAAACGTCCGCAATGTGTTATTAAGTTGTCTAAGCGTCAATTTGTTTACACCACAATATATCCTGCCACCAGCCAGCCAACAGCTCCCCGACCGGCAGCTCGGCACAAAATCACCACTCGATACAGGCAGCCCATCAGTCCGGGACGGCGTCAGCGGGAGAGCCGTTGTAAGGCGGCAGACTTTGCTCATGTTACCGATGCTATTCGGAAGAACGGCAACTAAGCTGCCGGGTTTGAAACACGGATGATCTCGCGGAGGGTAGCATGTTGATTGTAACGATGACAGAGCGTTGCTGCCTGTGATCAATTCGGGCACGAACCCAGTGGACATAAGCCTGTTCGGTTCGTAAGCTGTAATGCAAGTAGCGTATGCGCTCACGCAACTGGTCCAGAACCTTGACCGAACGCAGCGGTGGTAACGGCGCAGTGGCGGTTTTCATGGCTTGTTATGACTGTTTTTTTKGGGKACAGTCTATGCCTCGGGCATCCAAGCAGCAAGCGCGTTACGCCGTGGGTCGATGTTTGATGTTATGGAGCAGCAACGATGTTACGCAGCAGGGCAGTCGCCCTAAAACAAAGTTAAACATCATGGGGGAAGCGGTGATCGCCGAAGTATCGACTCAACTATCAGAGGTAGTTGGCGTCATCGAGCGCCATCTCGAACCGACGTTGCTGGCCGTACATTTGTACGGCTCCGCAGTGGATGGCGGCCTGAAGCCACACAGTGATATTGATTTGCTGGTTACGGTGACCGTAAGGCTTGATGAAACAACGCGGCGAGCTTTGATCAACGACCTTTTGGAAACTTCGGCTTCCCCTGGAGAGAGCGAGATTCTCCGCGCTGTAGAAGTCACCATTGTTGTGCACGACGACATCATTCCGTGGCGTTATCCAGCTAAGCGCGAACTGCAATTTGGAGAATGGCAGCGCAATGACATTCTTGCAGGTATCTTCGAGCCAGCCACGATCGACATTGATCTGGCTATCTTGCTGACAAAAGCAAGAGAACATAGCGTTGCCTTGGTAGGTCCAGCGGCGGAGGAACTCTTTGATCCGGTTCCTGAACAGGATCTATTTGAGGCGCTAAATGAAACCTTAACGCTATGGAACTCGCCGCCCGACTGGGCTGGCGATGAGCGAAATGTAGTGCTTACGTTGTCCCGCATTTGGTACAGCGCAGTAACCGGCAAAATCGCGCCGAAGGATGTCGCTGCCGACTGGGCAATGGAGCGCCTGCCGGCCCAGTATCAGCCCGTCATACTTGAAGCTAGACAGGCTTATCTTGGACAAGAAGAAGATCGCTTGGCCTCGCGCGCAGATCAGTTGGAAGAATTTGTCCACTACGTGAAAGGCGAGATCACCAAGGTAGTCGGCAAATAATGTCTAGCTAGAAATTCGTTCAAGCCGACGCCGCTTCGCGGCGCGGCTTAACTCAAGCGTTAGATGCACTAAGCACATAATTGCTCACAGCCAAACTATCAGGTCAAGTCTGCTTTTATTATTTTTAAGCGTGCATAATAAGCCCTACACAAATTGGGAGATATATCATGCATGACCAAAATCCCTTAACGTGAGTTTTCGTTCCACTGAGCGTCAGACCCCGTAGAAAAGATCAAAGGATCTTCTTGAGATCCTTTTTTTCTGCGCGTAATCTGCTGCTTGCAAACAAAAAAACCACCGCTACCAGCGGTGGTTTGTTTGCCGGATCAAGAGCTACCAACTCTTTTTCCGAAGGTAACTGGCTTCAGCAGAGCGCAGATACCAAATACTGTCCTTCTAGTGTAGCCGTAGTTAGGCCACCACTTCAAGAACTCTGTAGCACCGCCTACATACCTCGCTCTGCTAATCCTGTTACCAGTGGCTGCTGCCAGTGGCGATAAGTCGTGTCTTACCGGGTTGGACTCAAGACGATAGTTACCGGATAAGGCGCAGCGGTCGGGCTGAACGGGGGGTTCGTGCACACAGCCCAGCTTGGAGCGAACGACCTACACCGAACTGAGATACCTACAGCGTGAGCTATGAGAAAGCGCCACGCTTCCCGAAGGGAGAAAGGCGGACAGGTATCCGGTAAGCGGCAGGGTCGGAACAGGAGAGCGCACGAGGGAGCTTCCAGGGGGAAACGCCTGGTATCTTTATAGTCCTGTCGGGTTTCGCCACCTCTGACTTGAGCGTCGATTTTTGTGATGCTCGTCAGGGGGGCGGAGCCTATGGAAAAACGCCAGCAACGCGGCCTTTTTACGGTTCCTGGCCTTTTGCTGGCCTTTTGCTCACATGTTCTTTCCTGCGTTATCCCCTGATTCTGTGGATAACCGTATTACCGCCTTTGAGTGAGCTGATACCGCTCGCCGCAGCCGAACGACCGAGCGCAGCGAGTCAGTGAGCGAGGAAGCGGAAGAGCGCCTGATGCGGTATTTTCTCCTTACGCATCTGTGCGGTATTTCACACCGCATATGGTGCACTCTCAGTACAATCTGCTCTGATGCCGCATAGTTAAGCCAGTATACACTCCGCTATCGCTACGTGACTGGGTCATGGCTGCGCCCCGACACCCGCCAACACCCGCTGACGCGCCCTGACGGGCTTGTCTGCTCCCGGCATCCGCTTACAGACAAGCTGTGACCGTCTCCGGGAGCTGCATGTGTCAGAGGTTTTCACCGTCATCACCGAAACGCGCGAGGCAGGGTGCCTTGATGTGGGCGCCGGCGGTCGAGTGGCGACGGCGCGGCTTGTCCGCGCCCTGGTMGRTTGCCTGGCCGTAGGCCAGCCATTTTTGAGCGGCCAGCGGCCGCGATAGGCCGACGCGAAGCGGCGGGGCGTAGGGAGCGCAGCGACCGAAGGGTAGGCGCTTTTTGCAGCTCTTCGGCTGTGCGCTGGCCAGACAGTTATGCACAGGCCRGGCGGGTTTTAAGAGTTTTAATAAGTTTTAAAGAGTTTTAGGCGGAAAAATCGCCTTTTTTCTCTTTTATATCAGTCACTTACATGTGTGACCGGTTCCCAATGTACGGCTTTGGGTTCCCAATGTACGGGTTCCGGTTCCCAATGTACGGCTTTGGGTTCCCAATGTACGTGCTATCCACAGGAAAGAGACCTTTTCGACCTTTTTCCCCTGCTAGGGCAATTTGCCCTAGCATCTGCTCCGTACATTAGGAACCGGCGGATGCTTCGCCCTCGATCAGGTTGCGGTAGCGCATGACTAGGATCGGGCCAGCCTGCCCCGCCTCCTCCTTCAAATCGTACTCCGGCAGGTCATTTGACCCGATCAGCTTGCGCACGGTGAAACAGAACTTCTTGAACTCTCCGGCGCTGCCACTGCGTTCGTAGATCGTCTTGAACAACCATCTGGCTTCTGCCTTGCCTGCGGCGCGGCGTGCCAGGCGGTAGAGAAAACGGCCGATGCCGGGATCGATCAAAAAGTAATCGGGGTGAACCGTCAGCACGTCCGGGTTCTTGCCTTCTGTGATCTCGCGGTACATCCAATCAGCTAGCTCGATCTCGATGTACTCCGGCCGCCCGGTTTCGCTCTTTACGATCTTGTAGCGGCTAATCAAGGCTTCACCCTCGGATACCGTCACCAGGCGGCCGTTCTTGGCCTTCTTCGTACGCTGCATGGCAACGTGCGTGGTGTTTAACCGAATGCAGGTTTCTACCAGGTCGTCTTTCTGCTTTCCGCCATCGGCTCGCCGGCAGAACTTGAGTACGTCCGCAACGTGTGGACGGAACACGCGGCCGGGCTTGTCTCCCTTCCCTTCCCGGTATCGGTTCATGGATTCGGTTAGATGGGAAACCGCCATCAGTACCAGGTCGTAATCCCACACACTGGCCATGCCGGCCGGCCCTGCGGAAACCTCTACGTGCCCGTCTGGAAGCTCGTAGCGGATCACCTCGCCAGCTCGTCGGTCACGCTTCGACAGACGGAAAACGGCCACGTCCATGATGCTGCGACTATCGCGGGTGCCCACGTCATAGAGCATCGGAACGAAAAAATCTGGTTGCTCGTCGCCCTTGGGCGGCTTCCTAATCGACGGCGCACCGGCTGCCGGCGGTTGCCGGGATTCTTTGCGGATTCGATCAGCGGCCGCTTGCCACGATTCACCGGGGCGTGCTTCTGCCTCGATGCGTTGCCGCTGGGCGGCCTGCGCGGCCTTCAACTTCTCCACCAGGTCATCACCCAGCGCCGCGCCGATTTGTACCGGGCCGGATGGTTTGCGACCGCTCACGCCGATTCCTCGGGCTTGGGGGTTCCAGTGCCATTGCAGGGCCGGCAGACAACCCAGCCGCTTACGCCTGGCCAACCGCCCGTTCCTCCACACATGGGGCATTCCACGGCGTCGGTGCCTGGTTGTTCTTGATTTTCCATGCCGCCTCCTTTAGCCGCTAAAATTCATCTACTCATTTATTCATTTGCTCATTTACTCTGGTAGCTGCGCGATGTATTCAGATAGCAGCTCGGTAATGGTCTTGCCTTGGCGTACCGCGTACATCTTCAGCTTGGTGTGATCCTCCGCCGGCAACTGAAAGTTGACCCGCTTCATGGCTGGCGTGTCTGCCAGGCTGGCCAACGTTGCAGCCTTGCTGCTGCGTGCGCTCGGACGGCCGGCACTTAGCGTGTTTGTGCTTTTGCTCATTTTCTCTTTACCTCATTAACTCAAATGAGTTTTGATTTAATTTCAGCGGCCAGCGCCTGGACCTCGCGGGCAGCGTCGCCCTCGGGTTCTGATTCAAGAACGGTTGTGCCGGCGGCGGCAGTGCCTGGGTAGCTCACGCGCTGCGTGATACGGGACTCAAGAATGGGCAGCTCGTACCCGGCCAGCGCCTCGGMAVCCYCACCGCCGATGCGCGTGCCTTTGATCGCCCGCGACACGACAAAGGCCGCTTGTAGCCTTCCATCCGTGACCTCAATGCGCTGCTTAACCAGCTCCACCAGGTCGGCGGTGGCCCATATGTCGTAAGGGCTTGGCTGCACCGGAATCAGMACGAAGTCGGCTGCCTTGAWCGCGGACAYAGCCAAGTCCGCCGCCTGGGGCGCTMCGTCGATCACTACGAAGTCGCGCCGGCCGATGGCCTTCACGTCGCGGTCAATCGTCGGGCGGTCGATGCCGACAACGGTTAGCGGTTGATCTTCCCGCACGGCCGCCCAATCGCGGGCACTGCCCTGGGGATCGGAATCGACTAACAGAACATCGGCCCCGGCGAGTTGCAGGGCGCGGGCTAGATGGGTTGCGATGGTCGTCTTGCCTGACCCGCCTTTCTGGTTAAGTACAGCGATAACCTTCATGCGTTCCCCTTGCGTATTTGTTTATTTACTCATCGCATCATATACGCAGCGACCGCATGACGCAAGCTGTTTTACTCAAATACACATCACCTTTTTAGACGGCGGCGCTCGGTTTCTTCAGCGGCCAAGCTGGCCGGCCAGGCCGCCAGCTTGGCATCAGACAAACCGGCCAGGATTTCATGCAGCCGCACGGTTGAGACGTGCGCGGGCGGCTCGAACACGTACCCGGCCGCGATCATCTCCGCCTCGATCTCTTCGGTAATGAAAAACGGTTCGTCCTGGCCGTCCTGGTGCGGTTTCATGCTTGTTCCTCTTGGCGTTCATTCTCGGCGGCCGCCAGGGCGTCGGCCTCGGTCAATGCGTCCTCACGGAAGGCACCGCGCCGCCTGGCCTCGGTGGGCGTCACTTCCTCGCTGCGCTCAAGTGCGCGGTACAGGGTCGAGCGATGCACGCCAAGCAGTGCAGCCGCCTCTTTCACGGTGCGGCCTTCCTGGTCGATCAGCTCGCGGGCGTGCGCGATCTGTGCCGGGGTGAGGGTAGGGCGGGGGCCAAACTTCACGCCTCGGGCCTTGGCGGCCTCGCGCCCGCTCCGGGTGCGGTCGATGATTAGGGAACGCTCGAACKCGGCAATGCCGGCGAACACGGTCAACACCATGCGGCCGGCCGGCGTGGTGGTGTCGGCCCACGGCTCTGCCAGGCTACGCAGGCCCGCGCCGGCCTCCTGGATGCGCTCGGCAATGTCCAGTAGGTCGCGGGTGCTGCGGGCCAGGCGGTCTAGCCTGGTCACTGTCACAACGTCGCCAGGGCGTAGGTGGTCAAGCATCCTGGCCAGCTCCGGGCGGTCGCGCCTGGTGCCGGTGATCTTCTCGGAAAACAGCTTGGTGCAGCCGGCCGCGTGCAGTTCGGCCCGTTGGTTGGTCAAGTCCTGGTCGTCGGTGCTGACGCGGGCATAGCCCAGCAGGCCAGCGGCGGCGCTCTTGTTCATGGCGTAATGTCTCCGGTTCTAGTCGCAAGTATTCTACTTTATGCGACTAAAACACGCGACAAGAAAACGCCAGGAAAAGGGCAGGGCGGCAGCCTGTCGCGTAACTTAGGACTTGTGCGACATGTCGTTTTCAGAAGACGGCTGCACTGAACGTCAGAAGCCGACTGCACTATAGCAGCGGAGGGGTTGGATCAAAGTACTTTAAAGTACTTTAAAGTACTTTAAAGTACTTTGATCCCGAGGGGAACCCTGTGGTTGGCATGCACATACAAATGGACGAA
