# Supplementary material for: A modified Agrobacterium-mediated transformation for two oomycete pathogens
Source: PLoS Pathog. 2023 Apr 21;19(4):e1011346. doi: 10.1371/journal.ppat.1011346 (PMC10156060; doi:10.1371/journal.ppat.1011346)
Supplement: Attachment — Submitted filename: Response_to_Reviewers.docx [file ppat.1011346.s014.docx]

Response to Reviewers’ Comments

Dear editors and reviewers,

We thank you very much for the comments and suggestions. The comments and suggestions are valuable and very helpful for revising and improving our manuscript. We have made revisions according to the reviewers’ comments and suggestions, as described in the Response to Reviewers’ Comments.

Part I – Summary

**Reviewer#1:** In this MS, the authors claim to present a new and improved methodology of P. infestans transformation with Agrobacterium tumefaciens. In short, the authors show that expressing a host factor (AtVIP1) in Agrobacterium, fused with a Type IV translocation signal, enhances transformation efficiency in P. infestans and a series of recalcitrant (crop) species. Importantly, they show that this improved AMT method can be employed to target and modify P. infestans genes, opening up new avenues for tool development in

Overall, the authors present the data clearly and logically. The figures are well-constructed, and the data is of high quality (some comments on the figures are listed below). There are, however, issues with this MS that I have listed below.

**Major Comments:**

Whilst the work is important and novel, the advances made are limited. AMT is an established protocol in Phytophthora, and this system (including gene knockout) works in other Phytophthora pathogens (P. palmivora, Tian et al., 2020). The central crux of this work is that the authors claim to have a more efficient transformation system when AtVIP1 is expressed and deployed by Agrobacterium. The questions thus are: (i) Are the claims justified, and (ii) do the claims warrant publication in this journal?

In short, i think the conclusions are justified though I am not convinced that the advance represents a major leap forward.

**Response:** We appreciate Reviewer#1’s comments and constructive advice on our manuscript. Now a revised manuscript is submitted to address the concerns of Reviewer#1. We agree that it is more suitable to define our modified AMT method as an “option to be considered” for *P. infestans* transformation and precise genome editing. To further highlight the significance of our study, we have successfully applied our modified AMT method in transformation of *Plasmopara viticola*, a non-culturable and obligate biotrophic oomycete species, and generated a GFP-positive transformant. We believe these data have significantly improved our manuscript and hope their addition would suffice to meet the publication standard of *PLOS Pathogens.*

**Reviewer#2:** The main new discovery of this paper is that inclusion of a helper plasmid expressing AtVIP1 increases agrotransformation rates of Phytophthora infestans by about ten-fold. VIP1 is a plant bZIP protein shown previously to be involved in Agrobacterium-mediated transformation (AMT) in plants. Otherwise, the P. infestans transformation system described here is adapted from a method used by Vijn and Govers in 2003 in P. infestans, Phytophthora palmivora, and Pythium ultimum, and which was later adapted by others to P. sojae and in modified form to P. palmivora.

A survey of the literature identified four methods for transforming Phytophthora: protoplast transformation, zoospore electroporation, AMT, and microprojectile bombardment. It is impossible to say which is best, since the number of laboratories involved are limited and several different species are involved. Suffice it to say that the community benefits from having options.

In this paper, the authors first test the concept of using a VIP1 helper plasmid. First, they demonstrate that a VIP1-VirF-gfp fusion containing a type 4 secretion sequence (T4SS) was translocated into Nicotiania. Then, they demonstrated that adding this construct increased transient expression of GUS by about 10-fold in wheat, a species that is known to be difficult to transform. They then apply the method to P. infestans, obtaining data indicating that up to a 10-fold increase in transformation efficiency was achieved. Then using AMT with the helper plasmid and a CRISPR-Cas12a construct similar to that described previously for P. infestans (reference 12), they edit a MADS-box transcription factor (suppressing sporulation) and RXLR gene Avr8 (allowing evasion of resistance gene R8). The phenotypes resulting from editing the MADS gene are as expected since a paper from 2019 described the same phenotype in P. infestans using homology-based silencing. The Avr8 result is consistent with results from genetic segregation and agroinfiltration studies that demonstrated its interaction with R8.

My overall impression of the paper: The modified AMT method is a useful addition to the existing methods for P. infestans transformation. The CRISPR-Cas12a results are nice to see but not remarkable based on the prior paper on Cas12a in P. infestans, and the prior publications on the function of MADS and Avr8, although it is nice to see the Avr8-R8 interaction supported by a third type of evidence (adding to the segregation and agroinfiltration studies). Depending on what numbers you refer to, this either increases transformation rates 10-fold (based on data in the paper) or 4-fold (based on comparison to the existing AMT paper for P. infestans. Whether the modest increase claimed is sufficient for publication in PLOS Pathogens is an editorial decision. It is not clear if the method is more efficient than other transformation methods. However, I recognize that there could be major impact of the VIP1 helper if applied to other oomycetes and non-oomycetes as well.

**Response:** We really appreciate that Reviewer#2 recognizes the importance of our work while pointing out the shortcomings of our original submission. We have significantly revised our manuscript taking into account the feedback from Reviewer#2. In particular, we adapted our modified AMT method to a difficult-to-transform oomycete species *Plasmopara viticola*, and generated a GFP-positive transformant. We hope the revised version of our manuscript answers the queries raised by Reviewer#2*.*

**Reviewer#3:** Wang and colleagues report an Agrobacterium-mediated transformation and genome editing system for the Irish potato famine pathogen. This study comes after two publications on the same topic (Vijn & Govers, reference 7; Wu et al., reference 20). While the manuscript explores an interesting idea that has the potential to optimise the genetic manipulation of Phytophthora infestans, it lacks a comprehensive evaluation of transformation efficiency and a careful comparison to other methods using different P. palmivora strains.

**Response:** Reviewer#3’s constructive comments are encouraging and pointing out how to improve this manuscript. We have now conducted a more careful comparison between our improved AMT method and the conventional methods. Meanwhile, we have successfully applied this optimized protocol for the purpose of transforming a very challenging obligate plant pathogen, *Plasmopara viticola*, that causes grape downy mildew (figure 3). We hope these new data would significantly improve our manuscript and satisfy the Reviewer*.*

Part II – Major Issues: Key Experiments Required for Acceptance

**Reviewer#1:** In my view, the presence of pV1F does have a demonstrable impact on transformation rates. Whilst the primary gene constructs may have an effect (CAS12a transformation experiments seem to have lower yields), there is a consistent and significant positive impact on transformation outcomes.

**Response:** Thanks for your nice comments. We have now added more data analysis and performed additional experiments.

What is not clear, however, is whether there is a tangible effect on the number of transformants that express the gene of choice (GFP or CAS12a).

**Response:** Thanks to Reviewer#1 for this suggestion. We have added the details of transformants expressed *gfp* in Table 1 and line 219-227, 386-393. Indeed, not all positive transformants surviving on G418 showed a detectable GFP signal. As shown in Table 1, up to 74 G418 resistant JH19 colonies (transformed with pLY40-*gfp* and pV1F) were acquired in three transformation experiments, 34 of which showed a detectable GFP signal. In addition, up to 32 G418 resistant HB1501 colonies (transformed with pLY40-*gfp* and pV1F) were acquired in three transformation experiments, 14 of which showed a detectable GFP signal. Another G418 resistant 61 JH19 colonies (transformed with pLY40-*Cas12a-gfp* and pV1F) were acquired in three transformation experiments, 22 of which presented a detectable GFP signal. Finally, up to 22 G418 resistant HB1501 colonies (transformed with pLY40-*Cas12a-gfp* and pV1F) were acquired in three transformation experiments, 13 of which presented a detectable GFP signal.

Similarly, it is unclear whether improved transformation efficiency leads to a higher proportion of transformants carrying multiple inserts (resulting from successful integration into the chromosome). These characteristics (expression, integration, and insert number) are pertinent to transformation experiments and subsequent (genetic) analyses. Yet, the authors do not report on these features. I think they should; as with that data, the MS would greatly interest the reader.

**Response:** We thank Reviewer#1 for raising these good questions. We have performed western blot analysis on different transgenic lines (Supplementary figure 4), and Southern blot analysis was also performed to check the number of inserts (Figure 2f). Briefly, 14 GFP-signal colonies and 14 non-GFP-signal colonies were randomly selected. Western blot analysis suggested that a GFP-size protein could be detected in all 14 GFP-signal isolates, but not in the 14 non-GFP-signal strains (Supplementary figure 4). Results of the Southern blot analysis revealed that T-DNA fragments with the *nptII* gene are integrated into the genomic DNA of all six selected GFP-signal positive isolates and the non-GFP-signal transformant T1 (Figure 2f). T1, T4, T18, T21 and T25 showed a single band, while T11 and T35 showed two bands on the blot, suggesting that application of our modified protocol does not result in more transgene insertion events in the genome as compared to previous reports (Vijn and Govers, 2003). We hope these additional data meet the requirements by Reviewer#1 and provide more details on our research.

**Reviewer#2:** The paper would have a much greater impact if the concept of using the VIP1 helper plasmid was extended to other species, including fungi where AMT is common but not always efficient. Such information could be a real breakthrough for many pathogen species.

(One could also argue that the use of the VIP1 helper plasmid to enhance transformation in wheat is of equal, if not greater, impact; it is somewhat odd to see those results mixed in with the Phytophthora data but inasmuch as that data are shown as Supplementary Figures I do not object. I am surprised that this approach was not tested earlier in many plant species. I could only find a few papers on the subject, including reference 26 which describes how VIP overexpression increased transformation rates in Nicotiana. Perhaps dicot bZIPs are conserved such that orthologues can provide a VIP1-like activity, whilst monocots are more diverged.)

**Response:** We appreciate Reviewer#2’s enthusiasm about our work. Thanks for the detailed comments and suggestions on how to improve our manuscript. We have prepared a revised version of the manuscript taking into account Reviewer’s concerns. We have addressed all queries below.

For a paper that focuses mostly on methods, the transformation protocol was lacking in detail. These are some of the examples: Methods is fairly silent on how zoospores were generated and only in the Discussion are the actual amounts of zoospores used mentioned. The age of the cultures used to generate zoospores were 7 to 14 days in line 430, but 14 days in Fig S3. Is one better than the other? Rather than giving the details, we are just told that "the proper antibiotics" were used (li 432). Centrifugation speeds/g-forces were not provided. Agar concentrations in the overlay were not described. Also, whilst Methods says that Nytran was used, Fig. S3 alternates between Nytran and Hybond N+. Is there is difference?

**Response:** We thank Reviewer#2 for pointing out these issues with our manuscript. We have added more details to the Methods section, including:

**1**) In the revised manuscript, we provided details of the method we used for zoospore induction, and the actual numbers of zoospores we used in this study (Line 527-532).

**2)** We have corrected the age of *P. infestans* culture we used for zoospore induction, the average age is 14 days (Line 528).

**3)** We have added the details of antibiotics we used for Agrobacterium culture (Line 534) and description of the centrifugation speed we used (Line 536, 541).

**5)** Sorry, it was a typo. Now, we have corrected “Hybond N+ membrane” to “Nytran membrane” (Line 542 and Supplementary figure 3). We only use the Nytran membrane in our assays.

Whilst the paper does convincingly show that the helper plasmid raises transformation rates, the transformants themselves are poorly characterised. Perhaps this is not necessary here since there have been several papers on AMT in P. infestans and relatives already. However, as cited on li 345 about 1/3 of AMT events in Arabidopsis result in multiple integration events, so should not issues like this also be tested here? It would also be useful to address the issue of heterokaryons or mixtures arising from the method.

**Response:** We thank Reviewer#2 for raising the issue of insufficient characterization of transformants in this manuscript. This is a very constructive point. We have performed additional experiments and now included Southern blot analysis in the revised manuscript to show the integration events, obtained using the modified AMT method, in the representative *P. infestans* HB1501 transformants (Figure 2f) and western blot analysis of a number of transgenic lines (Supplementary figure 4). We have also discussed the potential heterokaryon issue (Line 402-405).

Since the authors are promoting their AMT method as an improvement over other transformation methods, could they report on the number of their GFP transformants that expressed easily visible levels of fluorescence, or the stability of expression? Low expression could be a problem with single-copy transformants.

**Response:** We thank Reviewer#2 for pointing out this issue with our study. We have now added the information on the numbers of transformants that showed a visible GFP signal detected by confocal microscopy (Table 1). Moreover, Figure 2f and Supplementary figure 4 demonstrate that transformants T4, T18 and T25 of HB1501, which displayed a detectable GFP signal (and a GFP-size band in the western blot), showed single bands in the Southern blot, while the transformant T1, which had no detectable GFP signal, also showed a single T-DNA integration event based on the same analysis. These additional data indicate that single-copy T-DNA integration events might not correlate with low expression of an exogenous gene when the AMT method is used in *P. infestans*. We hope these new data would fulfil the requirements by Reviewer#2 and provide more detail on the modified AMT protocol for *P. infestans*.

Considering that the authors are promoting their AMT method as an improvement over past AMT methods, could they discuss the variables that they tested as part of optimising their procedure?

**Response:** We thank Reviewer#2 for this good suggestion. After careful consideration, we decided it would be more appropriate to define our modified AMT method as an “option to be considered” for *P. infestans* transformation or gene editing. We have added a comparison between previously published protocols and our optimized AMT protocol for *P. infestans* as Supplementary Table 4.

**Reviewer#3:** - The central idea of this manuscript is to improve transformation rate by ectopic expression of a plant gene (AtVIP1) in A. tumefaciens. Conceptually, this is not new as it has been already used in various systems. For the specific purpose of oomycete transformation, the authors did not compare their method with the previously published AMT protocol for P. infestans. Instead, they report 61 and 22 transformants for two strains but the amount of zoospores is not mentioned in the M&M section, making it impossible to compare with the efficiency reported by Vijn & Govers. I would expect the authors to provide a comparison using PEG-protoelectroporation, AMT and AMT+AtVIP1.

**Response:**

We appreciate Reviewer#3’s constructive comments on our manuscript and recommendations. Yes, we agree with this reviewer that our work does not present a conceptual advance from a broad biological perspective. However, we have now significantly improved the description of the P. infestans transformation procedure, and extended application of our AMT procedure to a downy mildew pathogen. We have also toned down the significance of our improved AMT in the revised manuscript and added a comparison between previously published protocols and our optimized AMT method (Supplementary Table 4). In addition, we have given the details of the method we used for zoospore induction, and the actual numbers of zoospores we used in this study (Line 539-540, 560). We believe our protocol would make reverse genetics studies more feasible in oomycetes and accelerate the oomycete research in the future.

- On a similar note, the authors use sequence alignments (blast) to claim that oomycetes lack homologues of specific plant proteins important for AMT. Overall, this just reflects the phylogenetic distance between oomycetes and plants, and does not mean that the activities are absent from oomycete genomes. Did the authors consider domain-based screening and 3D structure modelling?

**Response:** Thank to Reviewer#3 for raising the suggestions of domain-based screening and 3D structure modelling. We understand these data would give additional details on AtVIP1-related domains in oomycete species. Actually, we have done some domain-based screening. For example, we have blasted the leucine-zipper domain of Arabidopsis VIP1 (KRAKRILANRQSAARSKERKIRYTGELERKVQTLQNEATTLSAQVTMLQRG) against the oomycete species database and got ‘No significant similarity found’ that was even worse than the outcome of the blast search using the full protein sequence. In addition, our key purpose in this study is to investigate the possibility of improving the AMT efficiency for *P. infestans* by involving a heterologous protein. Importantly, the fact that knocking-out VIP1 does not block AMT in Arabidopsis, suggests that AtVIP1 is helpful but is probably not essential for AMT in plant speices (Shi et al., *Plant J.* 2004). Figure 1a is here to illustrate that AtVIP1-like proteins are rare in oomycete species, which points out the possibility of utilizing AtVIP1 for improving AMT of *P. infestans*. We have also carefully revised our potentially overstated claims throughout manuscript.

- The sentence "The first AMT protocol of P. infestans was established in 2003 by Ah-Fong et al" (line 93) is incorrect. The first protocol was indeed established in 2003 but the correct reference is Vijn & Govers, Molecular Plant Pathology (doi: 10.1046/j.1364-3703.2003.00191.x). The reference number (7) at the end of the sentence is correct.

**Response:** Thanks to Reviewer#3 for pointing out this mistake, we have now corrected it (Line 115-117) in the revised manuscript.

- The effect of ectopic expression of AtVIP1 in A. tumefaciens on wheat transformation is not related to the manuscript main focus (P. infestans). As pointed out in the introduction, improvement of plant transformation by ectopic expression of plant genes in A. tumefaciens has already been established.

**Response:** We thank Reviewer#3 for the suggestion to remove the data on application of the modified AMT in wheat tissue. These data indicate the extensibility of our optimized AMT method, and we put them in Supplementary figure 2 already during the first round of submission. We have now also extended our modified AMT method to a difficult-to-transform oomycete *Plasmopara viticola*, and acquired one transformant expressing *gfp* (Figure 3). We hope these additional data meet the requirements by Reviewer#3 and provide more detail on our research.

Part III – Minor Issues: Editorial and Data Presentation Modifications

**Reviewer#1:** Minor comments:

Figure 1. The phylogenetic tree looks superficial and confuses me. Does the tree mean to depict evolutionary relationships of specific genes? If so, i am not clear which genes were used here and why the tree is branched the way it is. It is also unclear how the values were calculated (it is described in the methods and results, but I am unfamiliar with the method).

**Response:** We are thankful to Reviewer#1 for pointing out this problem. We are sorry for causing confusion on this part. In Figure 1a, the left phylogenic tree was generated by the OrthoFinder software based on variations in single-copy orthologous genes in released genomic data of different species, which indicates taxonomic status of all selected species. The right heatmap indicates similarity coefficient of known plant proteins involved in Arabidopsis AMT process and their homologues in selected plants and oomycete species. The similarity coefficient shown in figure 1a is derived via the ratio between the highest alignment score (bit-score) of local BLAST results from target species and the one from Arabidopsis. We have rearranged the description in Line 162-164 and legend of Figure 1.

Figure 2. Panel d: Please include a negative control (transformed with EV) to demonstrate that signal is specific to GFP (not fluorescence coming from mycelia). Panel e: Please have a coomassie stained gel as a loading control; indicate the (expected) size of the GFP band.

**Response:** We are thankful to Reviewer#1 for this suggestion. We have added a negative control Pi-isolate transformed with empty pLY40 to panel e of Figure 2 (previously Figure 2d). We have added Coomassie-stained gel as loading control and indicated the expected size of GFP bands in panel d of Figure 2 (previously Figure 2e).

In figure 3b, please add a non-GFP control and, for 3c, a loading control (as specified for fig 2).

**Response:** We are thankful to Reviewer#1 for this suggestion. We have added a negative control Pi-isolate transformed with empty pLY40 to panel b of Figure 4b (previously Figure 3b). We have added Coomassie-stained gel as loading control to panel c of Figure 4 (previously Figure 3c).

Finally, the text does have a fair number of spelling and grammatical errors. This MS should undergo a solid round of editing to ensure it meets publication standards.

**Response:** We are thankful to Reviewer#1 for raising the issue of writing errors in the manuscript. We have now polished English writing throughout the manuscript by a native speaker to ensure it meets the requirements of *PLOS Pathogens.*

**Reviewer#2:** In Introduction and Discussion, the authors make many statements about problems with the existing methods for transformation. The authors are pleading why their method is a major improvements, but their arguments are too strong, not always justified, and include errors. For example:

Line 66: Transformation rates cited in papers 20 or 30 years ago ("0.1 to 2 per microgram") may not hold today as improvements have likely been made over time including different enzyme mixtures and osmotica. Moreover, there is no way to compare those rates with the AMT rates described here. The methods are totally different.

**Response:** We are thankful to Reviewer#2 for raising the issue of overstating in our manuscript. It is indeed not appropriate to make comparisons between our data and results in those reports. After careful consideration of suggestions from all Reviewers, we realized it would be more suitable to define our modified AMT method as an “option to be considered” for *P. infestans* transformation or gene editing. We have deleted this improper description through the manuscript.

Line 67: The statement that most transformants from protoplast transformation are heterokaryons is wrong. I could only find a statement in the literature saying that heterokaryons were NOT found. Furthermore, the current paper does not test for heterokaryosis.

**Response:** We are thankful to Reviewer#2 for pointing out our wrong description of heterokaryons during transformation. Indeed, we have no data to prove that the protoplast transformation would introduce the issue of heterokaryons. We have deleted this improper description of heterokaryons in the manuscript.

Line 67: References 5 to 7 are used to describe the protoplast transformation method of obtaining stable transformants. However#5 does not involve stable transformation whilst#7 involved AMT

**Response:** We are thankful to Reviewer#2 for pointing out the wrong citations. We have rearranged our description and citations in Line 87-91.

Li 68: The statement that "Microprojectile projectile bombardment and zoospore electroporation seem feasible, but a low transformation efficiency still restrains related research (8,9)." This is odd since the number of transformants in paper#8 exceeds those in the current paper. The number of transformants from the optimised protocol in paper#9 is nearly identical to those described in the current paper.

**Response:** We are thankful to Reviewer#2 for pointing out this mistake. It is true that the comparisons between our modified AMT method and different methods in previous reports are incorrect and unnecessary. We have deleted these improper descriptions. We have also realized it would be more suitable to define our modified AMT method as an “option to be considered” for *P. infestans* transformation or gene editing.

Li 69: Whilst phenotypes may vary between transformants obtained in past studies, perhaps due to random integration sites, would not this also be true for AMT transformants? Furthermore, I did not see any data in the paper that thoroughly evaluated the phenotypes or integration events of the AMT transformants.

**Response:** We are thankful to Reviewer#2 for raising the issue of the lack of data on phenotypes and evaluation of integration events. We have added western blot and Southern blot analysis of representative *P. infestans* transformants (Supplementary figure 4 and Figure 2f). Briefly, the Southern blot analysis suggests that T-DNA fragments containing the *nptII* gene are integrated into genomic DNA of all 6 selected GFP-signal isolates and of the HB15101 transformant T1 that showed no GFP signal (Figure 2f). Also, the Southern blot results indicate that transformants T11 and T35 have two integrated T-DNA copies. These data suggest that T-DNA integration might not guarantee detectable expression of an exogenous gene in our study that is consistent with Reviewer#2’s point of view. We hope these additional data meet the requirements by Reviewer#2 and provide more detail on our research.

Li 77: Since the number of transformants in the Cas12 experiments described in reference 12 exceed that of the current AMT paper, it is not obvious that the transformation efficiencies from the protoplast method were low.

**Response:** We are thankful to Reviewer#2 for pointing out this mistake. We have deleted this improper description in the manuscript.

Li 320: The authors spend a lot of time presenting calculations that show that their method is 4 times more efficient than the AMT method described in ref. 7. Is this necessary? Please, a sentence focussed on the data in Table 1 would be sufficient.

**Response:** We are thankful to Reviewer#2 for making this good suggestion. We have deleted this improper description in the manuscript, and discussed the data presented in Table 1 (Line 386-390).

Li 331: The authors indicate that in the protoplast method, several problems might result including integration into an unstable site, degradation of the DNA by cellular enzymes. But would not similar events result from AMT? I am very familiar with AMT in plants, where T-DNA expression can also be unstable, or where only part of the T-DNA might integrate.

**Response:** We are thankful to Reviewer#2 for pointing out the wrong description of exogenous DNA integration during transformation of *P. infestans*. We agree with Reviewer#2 on the point that T-DNA integration during AMT could also be unstable, and have now changed the text accordingly (Line 382-386).

Li 336: Here again the authors appear to be claiming that their AMT method is less likely to result in heterokaryons than the protoplast method. Do the authors have data that show that many protoplasts used for transformation have multiple nuclei, resulting in heterokaryons? One could argue that heterokaryosis may be more common in AMT since transformation may occur as zoospores germinate and become multinucleate. Moreover, a fraction of P. infestans zoospores are multinucleate (for example, see Protoplasma 135:173, 1986). Whilst some papers may describe single-spore purification, this is just good microbiological practice to exclude mixed strains, and not just a way to eliminate potential heterokaryons. Based on the filter selection method shown in Fig. 2b I suggest that single-sporing should be part of the AMT protocol for P. infestans.

**Response:** We are thankful to Reviewer#2 for pointing out our wrong description of heterokaryons in this manuscript. We agree with Reviewer#2 that the heterokaryosis would also happen during the AMT procedure and our misleading discussions were unnecessary. The reference that Reviewer#2 mentioned (Oertel and Jelke, 1986) highlights that using zoospores of *P. infestans* might not eliminate the issue of heterokaryons during AMT in our study. We have deleted this improper description in the manuscript and added the single-sporing step to the protocol (Supplementary figure 3).

I suggest that Figure 1a be moved to Supplementary material. It is mostly relevant to studies of plants, not Phytophthora. One could also argue that for the broad readership of PLOS Pathogens, non-oomycetes should also be included. Nevertheless, I am not sure of the value of the figure it is not surprising that weak similarity was observed for VIP1 in oomycetes since transcription factors typically show little similarity between kingdoms outside of the DNA-binding domain, which represents only a small part of the protein.

**Response:** We are thankful to Reviewer#2 for this suggestion on Figure 1a. We totally agree that this manuscript is focused on AMT in oomycetes, while our key purpose in this study is to investigate the possibility of improving the AMT efficiency in *P. infestans* by involving a heterologous protein. Importantly, that fact that knocking-out VIP1 does not block AMT in Arabidopsis, indicates that AtVIP1 plays a role but is probably not essential for AMT (Shi et al., *Plant J.* 2004). Figure 1a here is to indicate that AtVIP1-like proteins are rare in oomycete species that points out the possibility of utilizing AtVIP1 for improving AMT in *P. infestans* strains. We sincerely ask Reviewer#2 to agree to keep Figure 1a in main results but it could be moved to supplementary data if Reviewer#2 insists on this.

I also did not like Fig. 1b. It does not add much to my understanding of the procedure, and seems out of context as it is introduced before the actual AMT experiments with P. infestans.

**Response:** We are thankful to Reviewer#2 for this suggestion on Figure 1b. Figure 1b presents the general idea of this manuscript that might help readers, especially the ones not familiar with AMT, to understand the key points of our modified AMT protocol. We sincerely ask Reviewer#2 to agree to keep Figure 1b in main results but it could be moved to supplementary data if Reviewer#2 insists on this.

Li 347: Whilst the use of A1A2 (self-fertile) strains of P. infestans to obtain biallelic edited strains through selfing is intriguing, have the authors tried this? Are A1A2 strains polyploid, making them difficult targets for gene editing? And are there data on how homogeneous phenotypes would be in the resulting strains?

**Response:** We are thankful to Reviewer#2 for raising the issue of self-fertility. We agree with Reviewer#2 that the discussion on mating-type is not necessary in the manuscript as we could not present supporting data at this moment. We have now adjusted the text accordingly.

Li 359: The paper that silenced the P. infestans MADS box gene is cited here in Discussion, but why was this not cited earlier instead of referencing the P. sojae MADS gene?

**Response:** We are thankful to Reviewer#2 for pointing out that the reference for the Pi-MADS gene is lacking in the manuscript prior to Discussion. We have now added the references for both *P. sojae* and *P. infestans* MADS-box genes in Line 269-270.

Li 375: This states that "no direct evidence has ever been presented" about whether Avr8 is involved in resistance mediated by R8. Direct evidence, however, is in the paper by Vossen et al (Theoretical and Applied Genetics 129: 1785, 2016). It is curious that this was not cited in the manuscript. A similar experiment confirming the interaction between Avr8 and R8 (by transient expression in Nicotiana was published in J. Exp Bot. 69, 1545, 2018. Avr8 (PITG_07558), under the name AvrSmira2, was also implicated as interacting with R8 based on segregation studies in reference 44.

**Response:** We are thankful to Reviewer#2 for point out our mistake concerning Avr8. We agree that the interaction between R8 and Avr8 has already been revealed and what we are saying is misleading. We have now deleted text with discussion of the above-mentioned point and added the references indicated by Reviewer#2 (Line 463-467).

On this topic, on li 373 did the authors mean "its responses to the R gene" instead of "its responses to the effectors."

**Response:** We are thankful to Reviewer#2 for pointing out this mistake. We have corrected this mistake (Line 462-463).

Fig. 4g: might the reduced lesion size in the MADS-edited strains be the result of fewer infection events, since sporangia were absent? Here infections were somehow done with mycelia, which is unnatural. Methods only provides details of how zoospore inoculations were performed.

**Response:** We are thankful to Reviewer#2 for pointing out this issue. We used the agar medium with mycelia for inoculation as MADS-box mutants produced few sporangia for zoospore induction. We have added the required information to the main text (Line 297-302) and the Methods section (Line 614-618).

Li 156: some explanation for the inclusion of VirF sequences would help the reader.

**Response:** We are thankful to Reviewer#2 for this suggestion. We have now added detailed information on VirF sequences in Line 182-184.

**Reviewer#3:**

line 48, 254: misspelled "avirulance"

**Response:** We are thankful to Reviewer#3 for pointing out this misspelling. We have corrected this mistake (Line 52, 311).

line 57: missing space

**Response:** We are thankful to Reviewer#3 for pointing out his mistake. We have corrected this mistake (Line 57).

line 103: "a set of proteins from host" maybe "a set of host proteins"?

**Response:** We are thankful to Reviewer#3 for pointing out this mistake. We have corrected "a set of host proteins" to "a set of proteins from host" (Line 124-125).

line 191: "in both two testedconstruct" missing space, and use either "two" or "both"

**Response:** We are thankful to Reviewer for pointing out the mistake. We have corrected the text accordingly (Line 219-220).
