# Supplementary material for: A modified Agrobacterium-mediated transformation for two oomycete pathogens
Source: PLoS Pathog. 2023 Apr 21;19(4):e1011346. doi: 10.1371/journal.ppat.1011346 (PMC10156060; doi:10.1371/journal.ppat.1011346)
Supplement: Attachment — Submitted filename: Response to Reviewers.docx [file ppat.1011346.s015.docx]

Response to Reviewers’ Comments

Dear editors and reviewers,

We would like to thank you for the time-consuming hard work on our submission. Your comments and suggestions are definitely valuable and lead us to significantly improve the manuscript. We have made revisions according to your comments and suggestions and described them in the Response to Reviewers’ Comments in detail.

Part I - Summary

Reviewer #1: This MS is a revised version and describes the impact of VIP1, a protein that, when expressed in eukaryote cells, enhances Agrobacterium tumefaciens-mediated transformation (AMT). The authors claim that the expression of VIP1 in A. tumefaciens leads to enhanced transformation rates in wheat, *P. infestans* and the obligate biotroph *Plasmopara viticola*.

The authors have made significant changes to the MS, including *P. viticola* transformation. The data is not overly convincing (one *P. viticola* transformant was presumably generated in one experiment), but considering the technical challenges associated with manipulating obligate biotrophs, this is a significant achievement. I am convinced that this approach will be tried and tested in other pathosystems, including obligate biotrophs.

Unfortunately, the authors opted not to include data describing cassette integration and copy number. While it would have been a nice addition, it does not diminish the overall findings and message of the work.

Taken together, this work represents a significant advance, possibly eliminating barriers that prevent transformation and gene editing in Eukaryote organisms considered recalcitrant to genetic transformation and modification.

**Response:** Thanks again to Reviewer#1 for encouraging comments and constructive suggestions on our manuscript. We believe there is still a lot of room for improvement of our modified AMT method for oomycete species, especially for *P. viticola.* We hope our manuscript attracts attention of scientists in related fields and, as a result, additional molecular toolboxes would be developed to facilitate transformation of hard-to-transform microorganisms. We are sorry for not being able to add more information on cassette integration and copy number to the manuscript at this stage, and we sincerely ask Reviewer#1 for the understanding on this issue. We have now submitted a revised manuscript based on all reviewers’ suggestions, and believe the manuscript has been further improved to meet the publication standard of *PLOS Pathogens.*

Reviewer #2: This is a revised version of a paper that mostly describes an improved Agrobacterium-mediated transformation procedure (AMT) for the plant pathogen Phytophthora infestans; the paper also goes on to demonstrate that the method can also be used to express a Cas12a protein for gene editing. That expression of the Arabidopsis VIP1 protein increased transformation frequencies in Phytophthora is convincing. I can see from the responses to reviewers that many improvements have been made compared to the original manuscript. It was disappointing that a test of the VIP1 strategy in fungi was not attempted, as this would have greatly increased the scope and impact of the paper, changing it from a quantitative improvement over the prior AMT method to something with broad application. Curiously, whilst the paper also reports that a similar strategy aided for wheat transformation--a finding that would have major impact--this is not mentioned in the Abstract. Nevertheless, it was exciting to see that the method apparently enabled the transformation of an obligate pathogen, the downy mildew Plasmopara viticola; this finding broadens the impact of the paper.

Whilst the paper is improved in many ways, there are still some problems and room for improvement. Issues related to the science are listed in points 1 to 17. The paper also needs improvement in some of the wording; some examples are shown after point 17.

**Response:** We are greatly honored to receive the positive feedback from Reviewer#2, and are really encouraged by the fact that Reviewer#2 recognizes the improvements of the manuscript done during the previous round of revisions. We also thank Reviewer#2 for their understanding on us not performing further AMT tests in fungi at this moment. Surely, we would like to extend our modified method to more microorganisms with transformation issues in the future. Although we have acquired a true *P. viticola* transformant with our modified AMT method in this study, the method needs to be further improved.

There are two reasons for us not to mention wheat transformation in the abstract. 1) We have only performed transient assays in wheat tissues, and improving transient expression efficiency does not guarantee an improved efficiency in making stable transgenic wheat lines. There is still a lot of work to do when it comes to improving the efficiency of generating stable transgenic wheat lines using AtVIP1. 2) As the manuscript is mostly focused on modifying the AMT method for oomycete pathogens, we decided to exclude the content on wheat from abstract to make sure we keep it succinct and to the point. We have answered all queries (points 1 to 17) raised by Reviewer#2 and also carefully corrected inappropriate wording throughout the whole manuscript. We hope these changes would meet the requirements of Reviewer#2 and publication standards of *PLOS Pathogens*.

Reviewer #3: This is a resubmission of a manuscript by Wang and colleagues about a modified Agrobacterium-mediated transformation for the Irish potato famine pathogen. The authors have made significant effort to address my concern. However, with the additions made in the revised version, I am now facing a dilemma: on one side, I am much more supportive to the manuscript. On the other side, I believe the Plasmopara viticola transformation is the most impactful finding reported in this study. It should be better emphasized and should be part of the title. I therefore expect the authors to reconsider the writing of their manuscript toward P. viticola transformation. I am also curious about possible toxicity effect of G418 on plant leaves. I would like to stress the point that such a protocol would be of invaluable interest for other researchers working on obligate biotrophs, including Bremia lactucae.

**Response:** We would like to express the sincerest gratitude to Reviewer#3 for their constructive comments which helped us improve this manuscript. We have now updated the title to underline applicability of the AMT method to *P. viticola* in the resubmitted version. While we understand the importance of acquiring a stable transformant of *P. viticola*, there are still several issues with the method at this stage. For instance, 1) the transformation efficiency is not high enough as compared to other oomycete species; 2) we have not performed a sufficient number of assays, involving other *P. viticola* isolates, to get a realistic estimate of the efficiency of *P. viticola* transformation using our method. We would like to ask for Reviewer#3’s understanding on the fact that we put emphasis on transformation on *P. infestans* in the manuscript. As to the issue of toxicity effect of G418 on plant leaves, we have not observed any negative effect on young grape leaves rinsed with geneticin G418 (5 mg/L) and Timentin (300 mg/L) and have added this information to the main text in Lines 632-634. We hope this improved manuscript version would satisfy Reviewer#3 and meet the standard of *PLOS Pathogens.*

Part II – Major Issues: Key Experiments Required for Acceptance

Reviewer #1: none

**Response:** We thank Reviewer#1 again for their hard work during the time-consuming reviewing process and approval of our last resubmission.

Reviewer #2: 1. Whilst I am glad to see a Southern blot used to characterize the nature of the transformation events, it is impossible to interpret the results since none of the plasmid maps show the full plasmids, location of HindIII sites, etc. Still, why do five of the seven transformants show the same lower band? (I can not interpret this result without maps and smaller size markers on the gel). It would also have been better to use the whole plasmid as a probe rather than npt2.

**Response:** We thank Reviewer#2 for the comments on our added data of Southern blot analysis. We have now added construct sequences of pLY40, pLY40-gfp and pLY40-Cas12a-gfp to the supplementary information and highlighted all HindIII restriction sites. We understand the advantage of using the whole plasmid as probe instead of the *nptII* sequence. However, using the full plasmid (with greater length and in much lower concentration compared to the PCR amplicon of *nptII*) would bring the risk of lower hybridization specificity and, consequently, false-negative results. For DIG-labeling, hybridization and chemiluminescence detection analysis, the recommended probe length is 300-1000 bp, which normally gives good results under most conditions. The mentioned lower bands in Figure 2f might be caused by non-specific hybridization or, potentially, by partial T-DNA integration events in selected transformants (also reported in plants: for example, Schouten et al., 2017, Plant Cell Reports). We have also added discussion regarding this comment in Lines 436-443, and believe have sufficiently clarified this point.

2. The results on Plasmopara viticola are important but preliminary, since the nature of the transformants (copy number, etc) are preliminary. As I think that transformation of this species is one of the more important parts of the paper, it is unfortunate that the results are so slim. Also, is it clear that the VIP1 plasmid was needed for transformation? This is not a trivial question considering that AMT without VIP1 has been used previously against many fungi include a powdery mildew, which like P. viticola is an obligate pathogen. Also, when the authors refer to the P. viticola transformants being stable for three generations, was this tested in the absence of selection. Methods does not make it clear how the stability test was performed.

**Response:** We thank Reviewer#2 for raising the issue of additional data on AMT for *P. viticola* in our last resubmission. We agree with the Reviewer that the presented data are preliminary. We have provided Southern blot analysis of the acquired *P. viticola* transformant in Figure 3d. We admit that our ability to apply the modified AMT method to biotrophic oomycetes is still limited. For instance, applying the AMT method to *P. viticola* in this study resulted in only one positive transformant. Although this transformant was acquired with our modified AMT method involving AtVIP1, we need to do more transformation attempts, involving additional *P. viticola* isolates, to draw a more solid conclusion in the future. In fact, we have performed more than 5 attempts to transform *P. viticola* without the helper plasmid pV1F and acquired no transformants showing G418 resistance. We would like to point out that in the manuscript we are only stating the fact that we obtained a positive *P. viticola* transformant with the modified AMT method, facilitated by AtVIP1, and we definitely wish to extend our protocol to other important microorganisms in the future. We have added the description of the method we used for acquiring sub-inoculation generations of the *P. viticola* transformant in Lines 634-636, and the definition of transformant stability in Lines 636-637.

3. Li 304. I am confused by the plant infection experiments with the MADS-edited strains. Methods states that the infections were performed using zoospores, but how could this be possible for strains that do not make sporangia? As written, it seems that infection did not occur but the mycelia grew poorly.

**Response:** We are sorry this part of the manuscript has caused confusion. We have now improved the description of how we performed these plant infection experiments with the *MADS-box* gene edited strains (Lines 324-326), which is consistent with the detailed description of the method in Lines 684-687.

4. Fig. 1b: This diagram may not represent how transformation is occurring. It seems unlikely that transformation is occurring on flagellated zoospores. The treatment conditions are likely to cause encystment of the zoospores followed by emergence of a germ tube. So the figure might be deceptive. This also raises the point of when transformation (DNA integration) is actually occurring. If this occurs after nuclear division begins post-germination, the transformants could be heterokaryons. Did the authors test this by passage through a single zoospore stage?

**Response:** We thank Reviewer#2 for pointing out the misleading information in Figure 1b. We agree that the encystment of zoospores might occur during transformation when zoospores are co-cultivated with Agrobacterium cells, and we have no evidence to suggest that the transformants were not heterokaryons. Actually, we observed no GFP signal in some of the progeny derived from the single zoospore stage GFP transformants of *P. infestans*. We thus reorganized Figure 1b taking into account Reviewer#2’s comments and also added related discussion in Lines 451-455.

Reviewer #3: No more experiment needed, but manuscript rewriting to be considered.

**Response:** We thank Reviewer#3 for recognizing the improvement in our last resubmission. We have made revisions according to all reviewers’ comments and put more emphasis on the AMT of *P. viticola* in the main text.

Part III – Minor Issues: Editorial and Data Presentation Modifications

Please use this section for editorial suggestions as well as relatively minor modifications of existing data that would enhance clarity.

Reviewer #1: none

**Response:** We thank Reviewer#1 again for recognizing the improvements in our last resubmission.

Reviewer #2: 5. Li 373 to 390: This part of Discussion needs to be improved. It talks about limitations of the protoplast method followed by limitations of AMT. But some of the points used for one could also apply to the other, such as the possibility of chimeric mycelia (there are no data to indicate that this is the case with AMT).

**Response:** We thank Reviewer#2 for bringing this issue to our attention. We have now rearranged the part of Discussion in question to better illustrate the limitations of both protoplast and AMT methods in the current version of the manuscript (Lines 405-423).

6. Li 213: do the authors mean the Bremia lactucae RPL41 promoter, or the P. sojae promoter (from whence the plasmids originated). On a similar topic, many of the components in the plasmids in Fig. 2 and elsewhere are not defined in the figure legends or main text.

**Response:** We thank Reviewer#2 for raising this issue. The *RPL41* promoter was originally derived from *P. sojae*. We have now added this information in Line 232 and also detailed all inserted components in Materials and Methods (Lines 556-580).

7. Li 208: Not clear what the authors mean by "poorly amenable, considering the many papers that describe the use of transformation. Also, references 6 and 31 do not address this issue.

The content of the first and much of the second paragraph of Discussion are odd. These paragraphs are focused on plant genetics, not the main topic of this paper (oomycetes).

**Response:** We thank Reviewer#2 for this suggestion. We have rewritten the corresponding text in Lines 227-228. We have also reorganized mentioned paragraphs of Discussion according to Reviewer#2’s advice (Lines 368-374 and 389-393).

8. Li 86. Both stable and transient silencing have been used.

**Response:** We thank Reviewer#2 for pointing this out. We have now rewritten the indicated sentence in Lines 96-98.

9. Li 108. It would be appropriate to describe the broad hosts against which AMT has been used; in the context of Plos Pathogens some mention of the extensive use of AMT against fungi would be appropriate.

**Response:** We thank Reviewer#2 for this suggestion. We have now added the information on applicability of *Agrobacterium* to a broad host range of non-plant organisms, and introduced some examples of AMT application in fungi (Lines 122-128) to fit better the scope of *PLOS Pathogens.*

10. Li 116. It is hard to understand the point being made about low numbers of zoospores in some strains. What the concentration of zoospores were in the prior AMT paper for P. infestans is unclear. Perhaps more importantly, It would be surprising that anyone would want to work on such strains since it would be hard to perform plant infections. Maybe the authors should instead focus on oomycete species that do not make zoospores?

**Response:** We thank Reviewer#2 for raising this issue. We have reorganized the paragraph in question and introduced some examples of oomycete species that produce few zoospores under artificial conditions (Lines 140-144).

11. Li 159. "Homologous" is misused. The approach is measuring similarity, not homology.

**Response:** We thank Reviewer#2 for this suggestion. We have changed ‘homologous proteins’ to ‘proteins with similarities’ in Lines 178.

12. Table 1: The headers would be less confusing if they said GFP+/G418r (adding the plus).

**Response:** We thank Reviewer#2 for this suggestion. We have added the plus after GFP in indicated headers of Table1.

13. Line 384. The discussion of methylation seems odd considering that the senior author (plus others) have previously reported a dearth of cytosine methylation in Phytophthora.

**Response:** We thank Reviewer#2 for this suggestion. We have removed the discussion on potential influence of DNA methylation on T-DNA inserts in *P. infestans* transformants as we agree that the dearth of cytosine methylation in *Phytophthora* species makes negative regulation of gene expression by DNA methylation, in T-DNA insert regions of obtained *P. infestans* transformants, unlikely.

14. Li 35: I question the use of the word "unique". Compared to what? One could say that anything is unique.

**Response:** We thank Reviewer#2 for pointing out the misuse of ‘unique’ in this sentence. We have now removed ‘unique’ from it (Line 34) to not cause ambiguity.

15. Li 36-38, 61-63. I question the use of "poorly understood". Whilst many questions remain, we are past the use of "poorly". Consider other wording such as "much remains to be learned."

**Response:** We thank Reviewer#2 for this suggestion. We have now corrected the sentence accordingly (Lines 36-37 and 62).

16. Li 70. I question the use of the word "non-culturable". It can be cultured on leaves. It would be more precise to say that it can not be cultured on axenic media.

**Response:** We thank Reviewer#2 for this suggestion. We have now replaced ‘non-culturable’ with ‘cannot be cultured on axenic media’ in Lines 71-72 and 83-84.

17. Li 79. Better to say "well-understood" rather than "well-studied"; the latter implies that people did not do a good job.

**Response:** We thank Reviewer#2 for this suggestion. We have now replaced ‘well-studied’ with ‘well-understood’ in Line 90 to avoid misunderstanding.

OTHER WORDING ISSUES

There are many poor word choices, grammar, or spelling in the paper, so the manuscript should be checked and improved. Here are just a few examples, as there were too many for me to list:

**Response:** We thank Reviewer#2 for pointing out the shortcomings of our writing in the last resubmission. We have carefully checked and revised the whole manuscript. We would like to express our sincere gratitude to Reviewer#2 for their contribution to the time-consuming manuscript review process.

Li 82. Did the authors really mean to use hysteretic?

**Response:** We thank Reviewer#2 for this suggestion. We have replaced ‘hysteretic genetic study’ with ‘reverse genetics studies’ to avoid potential misunderstanding (Line 95).

Li 90. Use of "open-minded" is wrong, but it is fine to say that we need to be open to new methods.

**Response:** We thank Reviewer#2 for this suggestion. We have replaced ‘open-minded’ with ‘we need to be open to new methods’, according to Reviewer#2’s advice, in Lines 101.

Li 165. Presumably the authors are using "similarity coefficient" in place of "sequence similarity", the latter of which is more commonly applied to BLAST.

**Response:** We thank Reviewer#2 for the suggestion. We have replaced ‘similarity coefficient’ with ‘sequence similarity’ in Lines 181, 184, 185, 187, 189, 191, 723, 725.

Li 453. Might be better to replace "might be required" to "could be used."

**Response:** We thank Reviewer#2 for this suggestion. We have replaced ‘might be required’ with ‘could be used’ in Line 510-511.

Author summary: many of the sentences have problems. For example, what "endogenously" means on line 66 is not clear. Also, Li 72: Has the POTENTIAL to accelerate. It has not yet done this.

**Response:** We thank Reviewer#2 for these suggestions regarding the author summary part. We have carefully revised this part in our current version of the manuscript.

As a result, we have replaced ‘endogenously express’ with ‘prokaryotic express’ in Lines 41 and 68 to make it clearer that in this case a plant gene (*AtVIP1*) is being expressed in *Agrobacterium*.

We used ‘potential’ in Line 75, as our modified AMT method has not yet been widely referred to in related studies at this moment.

Reviewer #3: The authors adequately addressed all my previous comments.

**Response:** We thank Reviewer#3 for recognizing the improvements in our last resubmission and appreciate their time-consuming work during the manuscript review process.
